# Molecular systematics of Chiritopsis-like *Primulina* (Gesneriaceae): one new species, one new name, two new combinations, and new synonyms

**DOI:** 10.1186/s40529-019-0266-x

**Published:** 2019-08-29

**Authors:** Wei-Bin Xu, Hsuan Chang, Jie Huang, Kuo-Fang Chung

**Affiliations:** 1 Guangxi Key Laboratory of Plant Conservation and Restoration Ecology in Karst Terrain, Guangxi Institute of Botany, Guangxi Zhuangzu Autonomous Region and Chinese Academy of Sciences, Guilin, 541006 China; 2 Research Museum and Herbarium (HAST), Biodiversity Research Center, Academia Sinica, Taipei, 11529 Taiwan; 3 School of Life Sciences, Fudan University, Shanghai, 200433 China

**Keywords:** Convergent evolution, Flora of China, Limestone cave flora, *Primulina chingipengii*, *Primulina subulata* var. *guilinensis*, *Primulina pseudoglandulosa*, Sino-Vietnamese limestone karst (SVLK), Taxonomy

## Abstract

**Background:**

The Gesneriaceae genus *Chiritopsis*, confined almost exclusively to cave or cave-like microhabitats of limestone karsts of southern China, was described to distinguish it from *Chirita* by much smaller flowers and generally miniature plant sizes in the former genus. However, molecular phylogenetic analyses showed that *Chiritopsis* is polyphyletic and its species delimitation has been problematic. To understand how many times Chiritopsis-like species have evolved from within the recircumscribed *Primulina* and to further clarify their species identification, we sampled all but two recently described species of Chiritopsis-like *Primulina* and reconstructed their phylogenetic relationship based on DNA sequences of nuclear ITS and chloroplast *trnL*-*F* and *trnH*-*psbA*.

**Results:**

With 182 accessions of 165 taxa of *Primulina* sampled, our analyses placed the 40 accessions of 25 taxa of Chiritopsis-like *Primulina* in 17 unrelated positions, indicating at least 17 independent origins of the traits associated with caves or cave-like microhabitats. Of the 17 clades containing Chiritopsis-like *Primulina*, Clade 1 is composed of *P. bipinnatifida*, *P. cangwuensis*, *P. jianghuaensis*, *P. lingchuanensis*, and *P. zhoui*, as well as additional samples that show variable and overlapping morphology in leaf shapes. Clade 10 includes *P. cordifolia*, *P. huangii*, and *P. repanda*, while *Primulina repanda* var. *guilinensis* is not placed within Clade 10. *Primulina glandulosa* var. *yangshuoensis* is not placed in the same clade of *P. glandulosa*.

**Conclusions:**

Based on our data, *P. cangwuensis*, *P. jianghuaensis*, and *P. lingchuanensis* are proposed to synonymize under *P. bipinnatifida*, with *P. zhoui* treated as a variety of *P. bipinnatifida*. *Primulina repanda* var. *guilinensis* is transferred as *P. subulata* var. *guilinensis* comb. nov. and *Primulina pseudoglandulosa* nom. nov. is proposed for *P. glandulosa* var. *yangshuoensis*. One new species is named *P. chingipengii* to honor the late Dr. Ching-I Peng (1950–2018).

**Electronic supplementary material:**

The online version of this article (10.1186/s40529-019-0266-x) contains supplementary material, which is available to authorized users.

## Background

The Gesneriaceae genus *Chiritopsis* W.T.Wang was established to distinguish it from *Chirita* Buch.-Ham. ex D.Don by the much smaller flowers, significantly shorter ovaries comparing to the styles, and generally smaller plant sizes in the former genus (Wang 1981). A total of 14 species and 3 additional varieties of *Chiritopsis* had been described (Table 1). Species of *Chiritopsis* inhabit caves or cave-like microhabitats on limestone karsts (Xu et al. 2012; Chung et al. 2014) or Danxia landforms (Shen et al. 2010) in Guangxi, Guangdong and adjacent regions of the Hunan Province (Li and Wang 2004; Wu et al. 2011), with *C. xiuningensis* disjunctly distributed in Anhui (Liu and Guo 1989), Zhejiang (Xia et al. 2011), and Fujian (Geng et al. 2014). Wang (1992) hypothesized that *Chiritopsis* and *Chirita* are sister groups, and the largely discontinuous and disjunct distributions of *Chiritopsis* likely reflect effects of Pleistocene glaciations.

**Table 1:** List of published names of Chiritopsis-like *Primulina* and references to its basionym. Specie names in bold denote accepted names of this study

Taxon	References
*Chiritopsis bipinnatifida* W.T.Wang [≡ ***Primulina bipinnatifida*** (W.T.Wang) Yin Z.Wang & J.M.Li]	Wang (1981)
*Chiritopsis confertiflora* W.T.Wang [≡ ***Primulina confertiflora*** (W.T.Wang) Mich.Möller & A.Weber]	Wang (1981)
*Chiritopsis cordifolia* D.Fang & W.T.Wang [≡ ***Primulina cordifolia*** (D.Fang & W.T.Wang) Yin Z.Wang]	Wang (1982)
*Chiritopsis danxiaensis* W.B.Liao, S.S.Lin & R.J.Shen [≡ ***Primulina danxiaensis*** (W.B.Liao, S.S.Lin & R.J.Shen) W.B.Liao & K.F.Chung]	Shen et al. (2010)
*Chiritopsis glandulosa* D.Fang, L.Zeng & D.H.Qin [≡ ***Primulina glandulosa*** (D.Fang, L.Zeng & D.H.Qin) Yin Z.Wang]	Fang et al. (1993)
*Chiritopsis glandulosa* var. *yangshuoensis* F.Wen, Y.Wang & Q.X.Zhang [≡ *Primulina glandulosa* var. *yangshuoensis* (F.Wen, Y.Wang & Q.X.Zhang) Mich.Möller & A.Weber ≡ ***Primulina pseudoglandulosa*** W.B.Xu & K.F.Chung]	Wen et al. (2008)
*Chiritopsis hezhouensis* W.H.Wu & W.B.Xu [≡ ***Primulina hezhouensis*** (W.H.Wu & W.B.Xu) W.B.Xu & K.F.Chung]	Wu et al. (2011)
*Chiritopsis jingxiensis* Yan Liu, W.B.Xu & H.S.Gao [≡ ***Primulina jingxiensis*** (Yan Liu, W.B.Xu & H.S.Gao) W.B.Xu & K.F.Chung]	Xu et al. (2009)
*Chiritopsis lingchuanensis* Yan Liu & Y.G.Wei [≡ *Primulina lingchuanensis* (Yan Liu & Y.G.Wei) Mich.Möller & A.Weber = ***Primulina bipinnatifida*** (W.T.Wang) Yin Z.Wang & J.M.Li]	Liu et al. (2006)
*Chiritopsis lobulata* W.T.Wang [≡ ***Primulina lobulata*** (W.T.Wang) Mich.Möller & A.Weber]	Wang (1982)
*Chiritopsis longzhouensis* B.Pan & W.H.Wu [≡ ***Primulina longzhouensis*** (B.Pan & W.H.Wu) W.B.Xu & K.F.Chung]	Pan et al. (2010)
*Chiritopsis mollifolia* D.Fang & W.T. Wang [≡ ***Primulina mollifolia*** (D.Fang & W.T.Wang) Yin Z.Wang]	Wang (1986)
*Chiritopsis repanda* var. *guilinensis* W.T.Wang [≡ *Primulina repanda* var. *guilinensis* (W.T.Wang) Mich.Möller & A.Weber ≡ ***Primulina subulata*** var. ***guilinensis*** (W.T.Wang) W.B.Xu & K.F.Chung]	Wang (1992)
*Chiritopsis repanda* W.T.Wang [≡ ***Primulina repanda*** (W.T. Wang) Yin Z.Wang]	Wang (1981)
*Chiritopsis subulata* var. *yangchunensis* W.T.Wang [≡ ***Primulina subulata*** var. ***yangchunensis*** (W.T.Wang) Mich.Möller & A.Weber]	Wang (1992)
*Chiritopsis subulata* W.T.Wang [≡ ***Primulina subulata*** (W.T.Wang) Mich.Möller & A.Weber]	Wang (1986)
*Chiritopsis xiuningensis* X.L.Liu & X.H.Guo [≡ ***Primulina xiuningensis*** (X.L.Liu & X.H.Guo) Mich.Möller & A.Weber]	Liu and Guo (1989)
*Primulina cangwuensis* X.Hong & F.Wen [= ***Primulina bipinnatifida*** (W.T.Wang) Yin Z.Wang & J.M.Li]	Hong et al. (2018)
***Primulina chingipengii*** W.B.Xu & K.F.Chung	This study
***Primulina cerina*** F.Wen, Yi Huang & W.Chuen Chou	Li et al. (2019)
***Primulina effusa*** F.Wen & B.Pan	Pan et al. (2017)
***Primulina huangii*** F.Wen & Z.B.Xin	Xin et al. (2018)
*Primulina jianghuaensis* K.M.Liu & X.Z.Cai [= ***Primulina bipinnatifida*** (W.T.Wang) Yin Z.Wang & J.M.Li]	Cai et al. (2014)
***Primulina maciejewskii*** F.Wen, R.L.Zhang & A.Q.Dong	Zhang et al. (2016)
***Primulina multifida*** B.Pan & K.F.Chung	Xu et al. (2012)
***Primulina niveolanosa*** F.Wen, S.Li & W.Chuen Chou	Li et al. (2019)
***Primulina pseudomollifolia*** W.B.Xu & Yan Liu	Xu et al. (2012)
*Primulina zhoui* F.Wen & Z.B.Xin [= ***Primulina bipinnatifida*** var. ***zhoui*** (F.Wen & Z.B.Xin) W.B.Xu & K.F.Chung]	Xin et al. (2018)

Although *Chiritopsis* appears to be a morphologically coherent group readily recognizable (Wang 1992; Wei et al. 2010), molecular phylogenetic analyses by Li and Wang (2007) showed that the genus (seven species sampled) is polyphyletic, nested within *Chirita* sect. *Gibbosaccus* C.B.Clarke. Subsequently, studies by Möller et al. (2009) and Wang et al. (2010) both showed that *Chirita* is also polyphyletic. To rectify the polyphyly, Wang et al. (2011) and Weber et al. (2011) proposed to abandon *Chirita*, transferring all its species to *Codonoboea* Ridl., *Damrongia* Kerr ex Craib, *Henckelia* Spreng., *Liebigia* Endl., *Microchirita* (C.B.Clarke) Yin Z.Wang, and *Primulina* Hance.

**Fig. 1:**
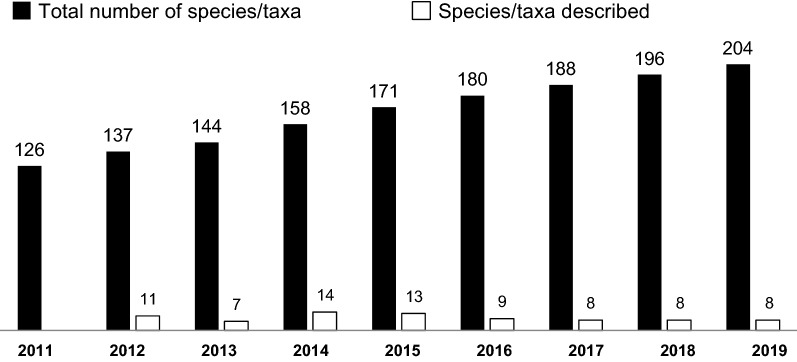
Number of *Primulina* species/taxa published since 2011 (Xu et al. 2017; Hong et al. 2019; Kong et al. 2019; Li et al. 2019; Wen et al. 2019; Ye et al. 2019)

Following the remodeling of *Chirita*, *Chiritopsis* and *Chirita* sect. *Gibbosaccus*, along with two species of *Wentsaiboea* D.Fang & D.H.Qin, were transferred to *Primulina* (Wang et al. 2011; Weber et al. 2011). With the inclusion of the ca. 130 species, *Primulina* is expanded drastically from a monotypic genus to one of the largest genera of the Old World Didymocarpoid Gesneriaceae and the largest genus of Chinese Gesneriaceae (Weber et al. 2013; Xu et al. 2017; Wen et al. 2019). Subsequent to its recircumscription, more than 70 new species have been added to *Primulina* (Fig. 1), with 10 species bearing *Chiritopsis*-like flowers reported from limestone karsts (Xu et al. 2012; Cai et al. 2014; Zhang et al. 2016; Pan et al. 2017; Hong et al. 2018; Xin et al. 2018; Li et al. 2019).

With 204 described taxa, the recircumscribed *Primulina* is the most species-rich plant genus on the vast limestone karst terrain stretching from southern China to northern Vietnam (Möller et al. 2016; Xu et al. 2017; Hong et al. 2019; Kong et al. 2019; Li et al. 2019; Wen et al. 2019). The diverse floral morphology and habitat preference of *Primulina* thus makes the genus an ideal clade to study evolutionary mechanisms that generated the high species diversity in the Sino-Vietnamese limestone karsts (Chung et al. 2014; Gao et al. 2015; Kong et al. 2017). The polyphyly of Chiritopsis-like *Primulina* indicates the homoplasious nature of the small corollas and generally lesser plant size characterizing the former *Chiritopsis*, suggesting multiple and independent origins of these traits. The highly polyphyletic Chiritopsis-like *Primulina* also suggests convergent evolution of the smaller corollas associated with their habitat preference (i.e., caves and cave-like microhabitat), reminiscent of the convergent evolution associated with pollination syndromes in the New World Gesneriaceae (e.g., Clark et al. 2012, 2015).

**Fig. 2:**
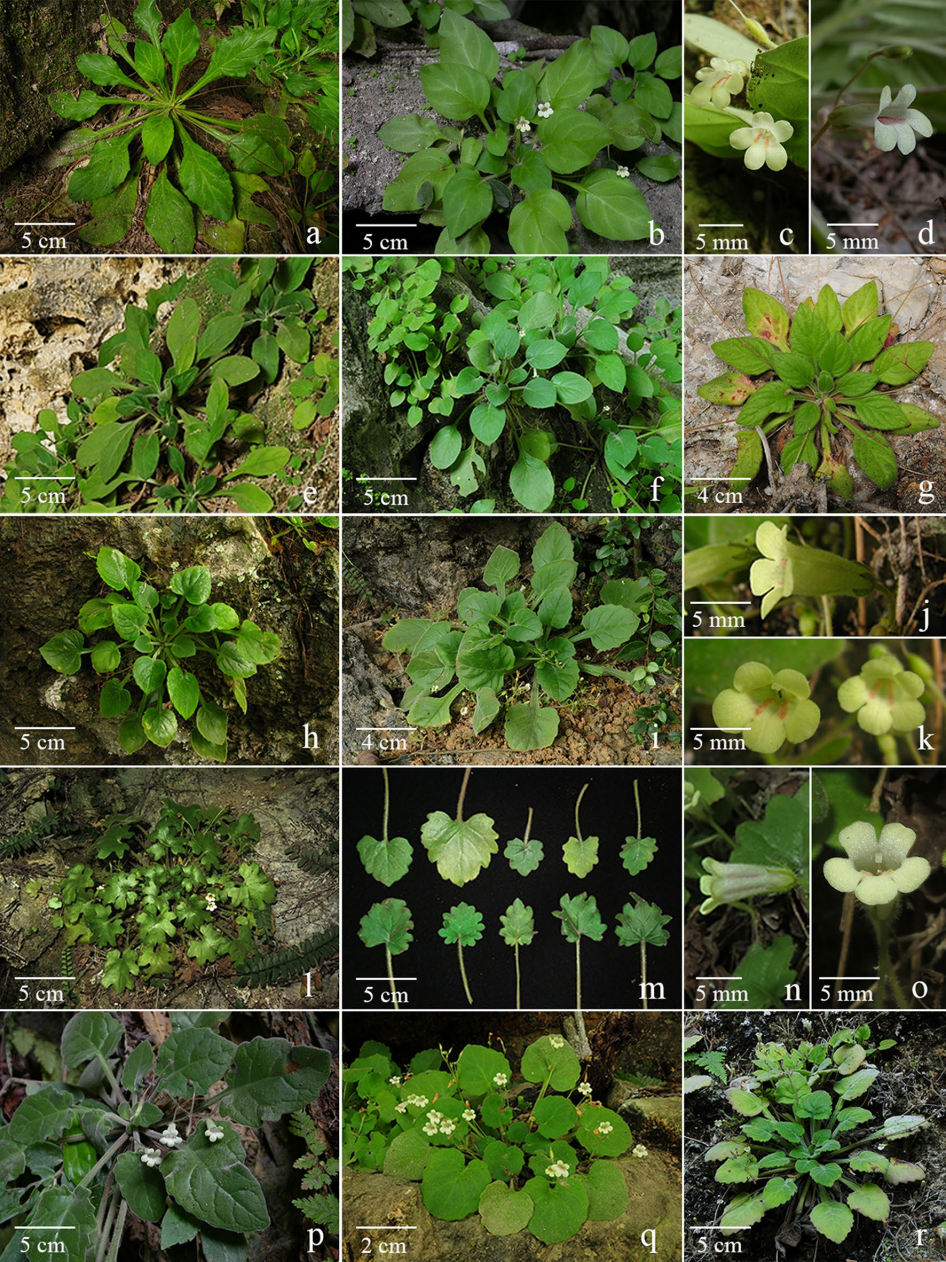
*Primulina repanda* (**a**–**g**), *P. cordifolia* (**h**–**k**, **p**–**r**), and *P. huangii* (**l**–**o**). **a**–**d** plants from the type locality of *Chiritopsis repanda* W.T.Wang (*Chung 1821*), **a**, **b** habit, **c** flowers (face view), **d** flower (side view); **e** plants from Liujiang (*Peng 22921*); **f** plants from Rongshui (*Chung 1815*); **g** a plant from Tian’e (*Chung* et al*. 1823*); **h**–**k** plants from the type locality of *C. cordifolia* W.T.Wang (*Chung 1817*), **h**, **i** habit, **j** flowers (side view), **k** flowers (face view); **l**–**o** plants from type locality of *P. huangii* F.Wen & Z.B.Xin, **l** habit, **m** variation in leaf shapes, **n** flower (side view), **o** flower (face view); **p** a plant from Bama (*Chung* et al*. 1828*); **q** plants from plants from Rong’an (*Chung* et al*. 1808*); **r** a plant from Donglan (*Chung* et al*. 1826*)

**Fig. 3:**
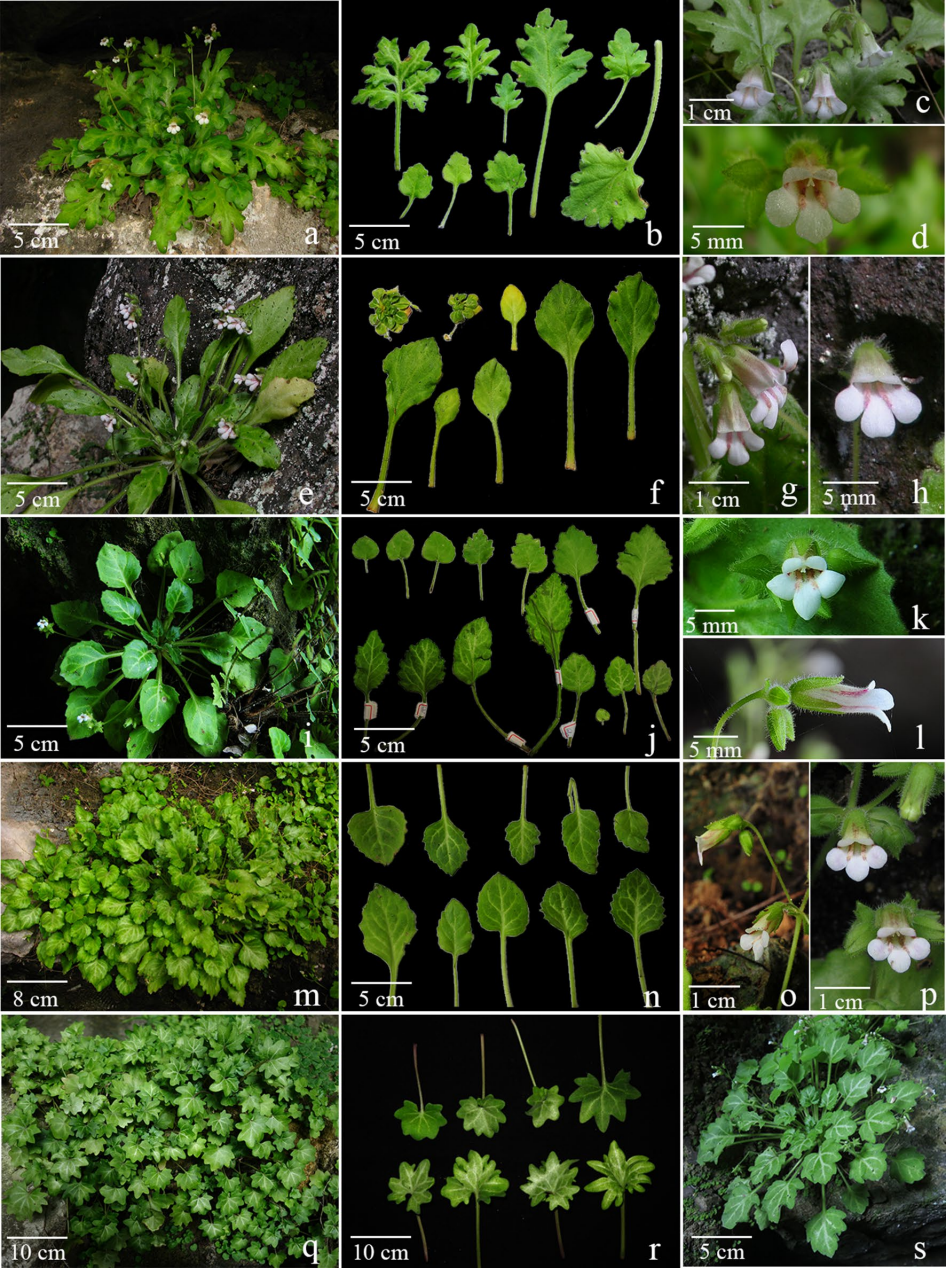
*Primulina bipinnatifida*. **a**–**d** plants from type locality of *Chiritopsis bipinnatifida* W.T.Wang (*Chung* et al*. 1863*), **a** habit, **b** variation in leaf shapes, **c** flowers (side view), **d** flower (face view); **e**–**h** plants from type locality of *C. lingchuanensis* Yan Liu & Y.G.Wei (*Chung 1802*), **e** habit, **f** variation in leaf shapes, **g** flowers (side view), **h** flowers (face view); **i**–**l** plants from type locality of *P. jianghuaensis* K.M.Liu & X.Z.Cai (*Chung 2932*), **i** habit, **j** variation in leaf shapes, **k** flower (face view), **l** flower (side view); **m**–**p** plants from type locality of *P. cangwuensis* X.Hong & F.Wen (*Chung 1842*), **m** habit, **n** variation in leaf shapes, **o** flowers (side view), **p** flowers (face view); **q**, **r** plants from type locality of *P. zhoui* F.Wen & Z.B.Xin (*F. Wen WF150718*-*01*), **q** habit, **r** variation in leaf shapes; **s** a plant from Lipu (*Chung 1852*)

Although *Primulina* is currently the most species-rich plant genus inhabiting the Sino-Vietnamese limestone karsts, the taxonomy and species delimitations of *Primulina* remain controversial (Wang et al. 2017; Yang et al. 2019), as Weber et al. (2011) has cautioned that “*It is also our impression that too many species have been described with numerous pairs or small groups of species growing in adjacent areas and differing only in slight, quantitative characters.*” For instance, *P. huangii* (Fig. 2l–o) and *P. zhoui* (Fig. 3q, r) were described based on their leaf morphology; however, both species have corollas identical to *P. cordifolia* and *P. repanda* (Fig. 2) and *P. bipinnatifida*, *P. lingchuanensis*, *P. jianghuaensis*, and *P. cangwuensis* (Fig. 3), respectively, that were overlooked by the author (Xin et al. 2018). Additionally, in the type localities of abovementioned species, we also observed considerable variation in leaf shapes (Figs. 2 and 3) apparently neglected and/or ignored by previous studies (Wang 1981, 1982; Liu et al. 2006; Cai et al. 2014; Hong et al. 2018; Xin et al. 2018). On the other hand, we notice two apparent cases of taxon misidentification of Chiritopsis-like *Primulina* in the GenBank (Vilgalys 2003). Specifically, the ITS sequence of *P. mollifolia* collected from the type locality used in current study (Xu et al. 2012; NCBI: JX506866) is very different from those of ‘*P. mollifolia*’ submitted by Li and Wang (2007; NCBI: DQ872847) and Kong et al. (2017; NCBI: KY394945). Instead, DQ872847 and KY394945 are identical to the ITS sequences of samples (JX506869 & JQ713837) collected from the type locality of *P. pseudomollifolia* (Xu et al. 2012). Actually, the misidentification of *P. mollifolia* also occurs in Wei et al. (2010)’s much cited book ‘Gesneriaceae of South China.’ Similar taxon identification is detected in *P. lingchuanensis* as the ITS of ‘*P. lingchuanensis*’ submitted by Kong et al. (2017; KY394922) is very different from the sequence generated from specimen collected from the type locality in current study (JX506914). Unfortunately, such GenBank misidentifications of both KY394922 (labeled as *P. lingchuanensis*) and KY394945 (labeled as *P. mollifolia*) have been transmitted in a recent publication by Ye et al. (2019). Regardless those taxon misidentifications, previous phylogenetic study by Kong et al. (2017) showed that two infraspecific taxa *P. glandulosa* var. *yangshuoensis* and *P. repanda* var. *guilinensis* are only distantly related to their respective parental binominal *P. glandulosa* and *P. repanda*, necessitating nomenclatural changes.

In our continuous efforts to investigate the diversity of the cave flora of Sino-Vietnamese karsts (Chung et al. 2014), we surveyed and collected in a considerable number of caves, including type localities of most species of former *Chiritopsis*, as well as species of Chiritopsis-like *Primulina* species described after the work of Wang et al. (2011) and Weber et al. (2011). To understand how many times the Chiritopsis-like *Primulina* species have evolved, we reconstructed up to date the most comprehensive phylogenetic relationship of *Primulina*, sampling 165 taxa (81% of existing taxa). Based on our analyses, we propose taxonomical changes to better reflect their phylogenetic relationships, including the recognition of a new species, two new name, a new combination, and three new synonyms.

## Methods

### Taxon sampling and DNA extraction and sequencing

A total of 117 accessions representing 103 taxa of *Primulina* (50.4%) were collected by the authors in southern China during 2009–2016, with additional samples provided by the Guangxi Institute of Botany in Guilin, China. Multiple accessions of Chiritopsis-like *Primulina* species with broader distributions (e.g., *P. bipinnatifida*, *P. cordifolia*, and *P. repanda*) were sampled to test species boundary. Our samples also included two unknown species of the Chiritopsis-like *Primulina*. Sixty-five species of *Primulina* with nuclear internal transcribed spacers (ITS) and *trnL*-*F* intron-spacer region sequences available in the Genbank were also included. We acknowledge the work of Kong et al. (2017) that sampled 199 populations of 159 described *Primulina* species using ten cpDNA regions and ten nuclear genes (including ITS). However, we did not include all DNA sequences of Kong et al. (2017) because (1) our data were sufficient to address our purpose (i.e., how many times Chiritopsis-like *Primulina* species have evolved) and (2) species identification of a majority of species in Kong et al. (2017) could not be confirmed because insufficient voucher information provided. A total of 182 accessions of *Primulina* representing 165 taxa (80.9%) were included (Additional file 1), with two species of *Petrocodon* chosen as outgroups based on previous studies (Wang et al. 2011; Weber et al. 2011). We sampled all but two recently described species of Chiritopsis-like *Primulina* [i.e., *P. cerina* and *P. niveolanosa*; Li et al. (2019)], including 40 accessions of 25 taxa (Table 1). Total genomic DNA was extracted from silica-gel dried leaves using the CTAB protocol (Doyle and Doyle 1987). DNA sequences of ITS and the chloroplast *trnL*-*F* intron-spacer region and *psbA*-*trnH* intergenic spacer were amplified based on the PCR procedures outlined in Möller et al. (2009) and Smissen et al. (2004).

### Molecular phylogenetic analyses

The DNA sequences were aligned using MUSCLE implemented in MEGA7 (Kumar et al. 2016) with subsequent manual adjustments. For those species with only ITS and *trnL*-*F* sequences, *psbA*-*trnH* sequences were treated as missing data. Substitution model test was selected using MrModeltest version 2.3 (Nylander 2004) for individual sequence matrix and concatenated matrix. The best-fit models under the Akaike Information Criterion (AIC) were GTR + G+I for ITS, GTR + G for *trnL*-*F*, GTR + G for *psbA*-*trnH*, and GTR + G+I for the concatenated matrix. Both sequence matrices were analyzed using maximum likelihood (ML) optimality criteria and Bayesian Inference (BI). The ML analyses with 1000 bootstrap resampling were conducted using RAxML-HPC (Stamatakis et al. 2008) via the CIPRES Portal (Miller et al. 2010), with a gamma model of rate heterogeneity and the substitution model GTR + G+I. Proportion of invariable sites was estimated by the program. The BI analyses were conducted with MrBayes version 3.2 (Ronquist et al. 2012). Four chains of Markov chain Monte Carlo were run for 5 million generations each and were sampled every 250 generations starting with a random tree. For each run, the first 25% of sampled trees were excluded as burn-in before Bayesian clade posterior probabilities and average branch lengths were calculated.

## Results and discussion

**Fig. 4:**
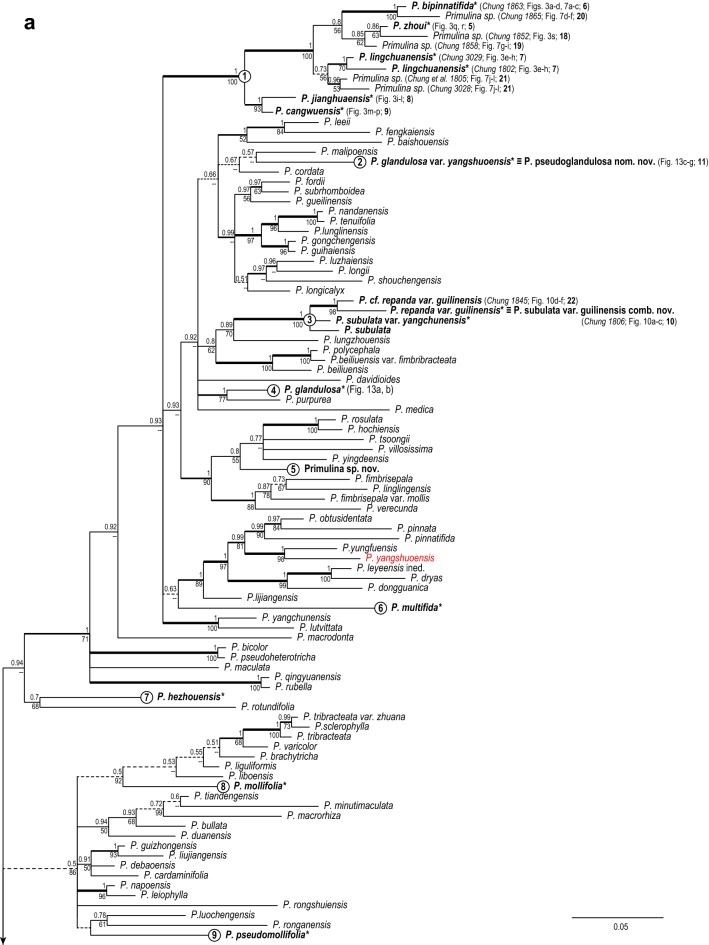

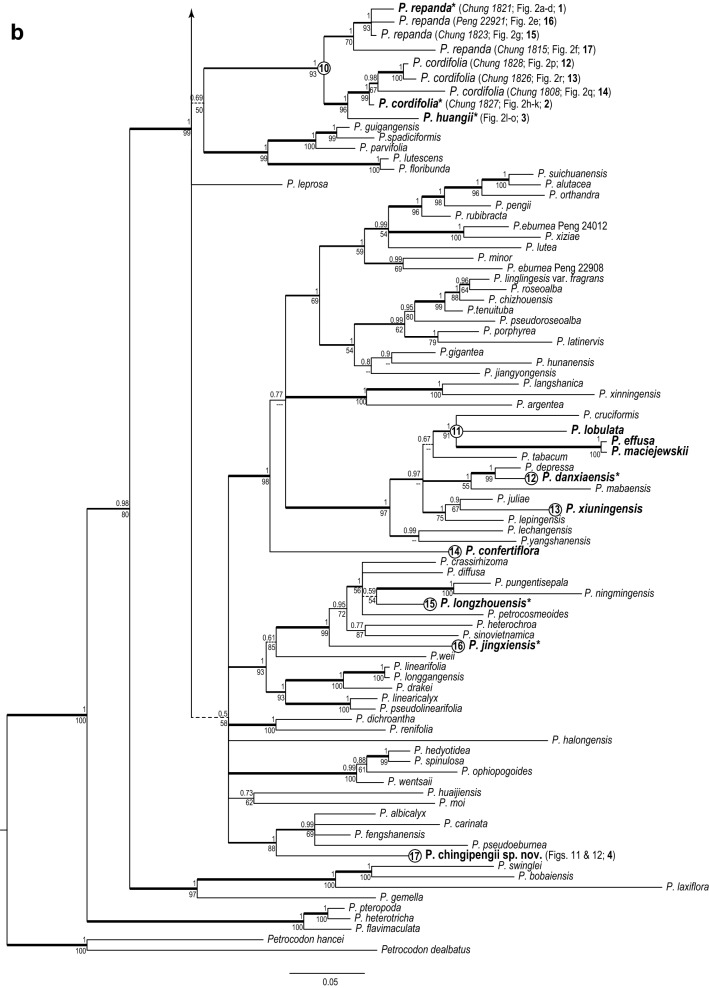
Bayesian majority-rule consensus tree with mean branch length based on the combined ITS and chloroplast (*trnL*-*F* and *trnH*-*psbA*) DNA sequences. Bayesian posterior probability (PP; > 0.50) and ML bootstrap support values (ML; > 50%) are shown above and below the branch around the corresponding node. Thickest clades denote both PP and ML higher than 0.95. Thick clades denote only PP > 0.50. Dashed branches denote clades with PP < 0.75. Species of Chiritopsis-like *Primulina* are shown in bold face, with * indicating materials from type locality. Numbered circles denote clades containing Chiritopsis-like *Primulina* species

The combined matrix of the three markers contained 3226 characters [ITS (184 sequences): 755 bp; *trnL*-*F* (182 sequences): 827 bp; *trnH*-*psbA* (112 sequences): 1644 bp]. Results of the Bayesian inferences and maximum likelihood were highly congruent, differing mainly in support values. The Bayesian majority-rule consensus tree with mean branch length is depicted in Fig. 4, annotated with bootstrap support values (BS) of the ML analyses and posterior probability (PP) values of BI analysis. Rooted by *Petrocodon*, the major clades identified in our phylogenetic analyses are basically congruent with previous studies (e.g., Guo et al. 2015; Kong et al. 2017; Ye et al. 2019), though relationships among these major clades remain poorly resolved. Nevertheless, our results are sufficient to address our main questions.

The phylogenetic analyses placed the 40 accessions of Chiritopsis-like *Primulina* in 17 unrelated positions (Fig. 4), indicating at least 17 independent origins of the small flowers and the habit. Because the Chiritopsis-like *Primulina* species are found almost exclusively in cave or cave-like environments (i.e., Chung et al. 2014), the homoplasious nature of those traits characterizing these habitat specialists suggest that strong selection and adaptation to the extreme habitat might have resulted in their convergent evolution (Pipan and Culver 2012; Trontelj et al. 2012). Further discussion on this issue will be dealt in our subsequent study.

### Clade 1

Clade 1 was composed of 11 accessions of Chiritopsis-like *Primulina* (Fig. 4a), including six accessions sampled from the type localities [*P. bipinnatifida* (*Chung 1863*), *P. cangwuensis*, *P. jianghuaensis*, *P. lingchuanensis* (*Chung* et al*. 1802* and *3029*), and *P. zhoui*], and five additional accessions (*Chung* et al*. 1805*, *1852, 1858*, *1865*, and *3028*) collected during our exploration in the limestone areas of Guangxi. Because uncertainty of species identification, those non-type accessions are labeled as “*Primulina sp.* (*voucher #;* Fig. #; **#** on map)”, such as “*Primulina sp.* (*Chung 1865*; Fig. 7d–f; 20)” for the accession sister *P. bipinnatifida* (Fig. 4a). In previous studies, the morphological similarity among the first four described species of Chiritopsis-like *Primulina* has been suggested as Liu et al. (2006) mentioned the similarity in floral morphology between *C. bipinnatifida* and *C. lingchuanensis*, Cai et al. (2014) compared *P. jianghuaensis* with *P. lingchuanensis* and *P. danxiaensis*, and Hong et al. (2018) differed *P. cangwuensis* from *P. repanda*, *P. subulata*, *P. jianghuaensis*, and *P. lobulata*.

**Fig. 5:**
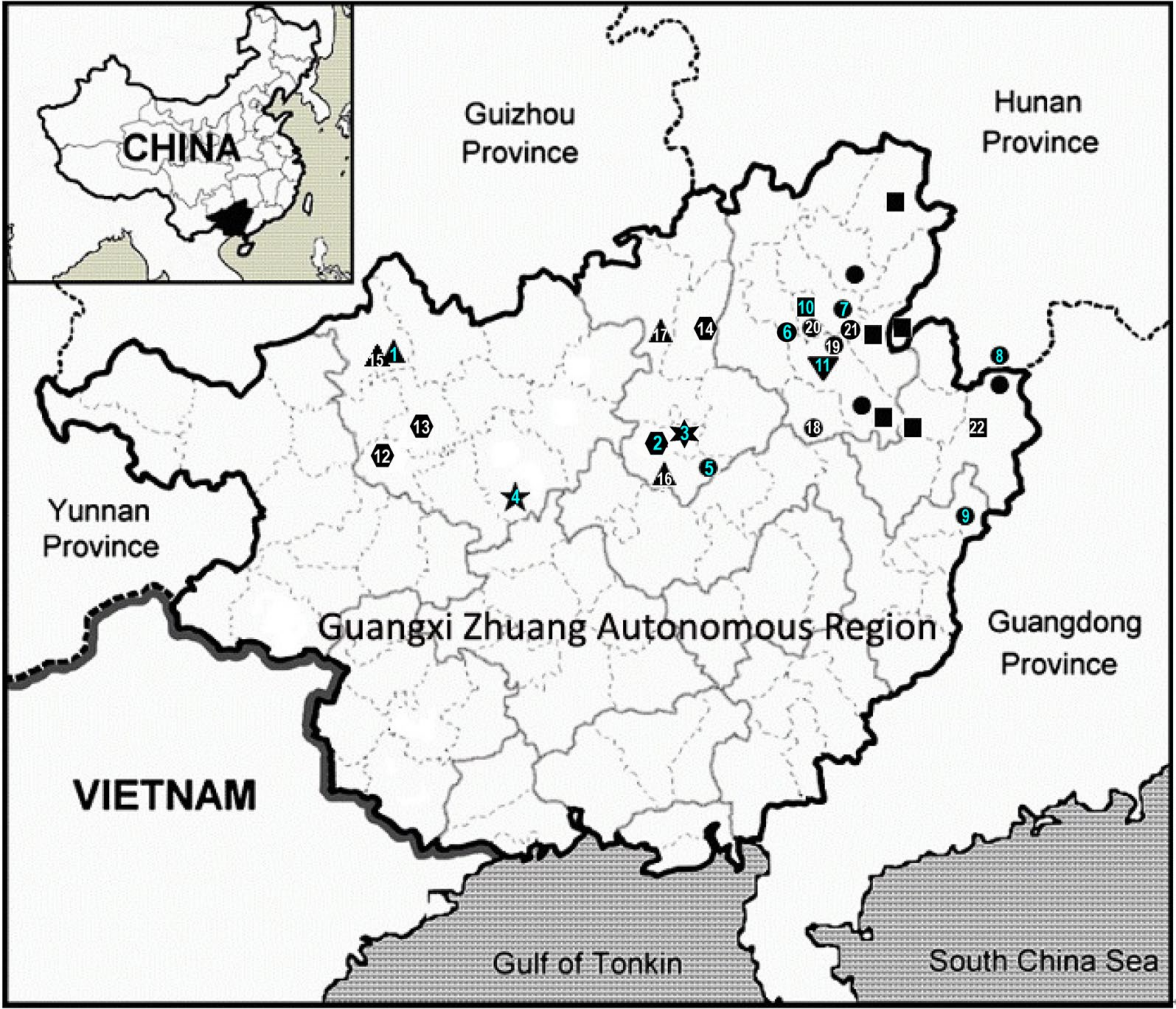
Distribution of *Primulina bipinnatifida* (black circles), *P. chingipengii* (black star), *P. cordifolia* (hexagons), *P. huangii* (hexagram), *P. subulata* var. *guilinensis* (black squares), *P. pseudoglandulosa* (black down-pointing triangle) and *P. repanda* (black up-pointing triangles) in Guangxi and Hunan, southern China. The type localities are marked by the light-blue numbers (1–11): 1. *Chiritopsis repanda*; 2. *C. cordifolia*; 3. *P. huangii*; 4. *P. chingipengii*; 5. *P. bipinnatifida* var. *zhoui* (≡ *P. zhoui*); 6. *C. bipinnatifida*; 7. *C. lingchuanensis*; 8. *P. jianghuaensis*; 9. *P. cangwuensis*; 10. *C. repanda* var. *guilinensis*; 11. *C. glanduola* var. *yangshuoensis*. Numbers 12 to 22 denote non-type localities sampled for current study: 12: Bama (*Chung 1828*); 13: Wuzhuang (*Chung 1826*); 14: Rong’an (*Chung 1808*); 15: Tian’e (*Chung 1823*); 16: Liujiang (*Peng 22921*); 17: Rongshui (*Chung 1815*); 18: Lipu (*Chung 1852*); 19: Yangshuo (*Chung 1858*); 20: Linggui (*Chung 1865*); 21: Lingchuan (*Chung 1805* and *3028*); 22: Hezhou (*Chung 2914*)

Within Clade 1, *P. jianghuaensis* and *P. cangwuensis* formed a strongly supported subclade (PP: 1; BS: 95) sister to a highly supported subclades (PP: 1; BS: 100) comprising the remaining accessions (Fig. 4a). Within the latter subclade, two accessions from the type locality of *P. lingchuanensis* (7 in Fig. 5) form a moderately supported clade (PP: 1; BS: 70) sister to a moderately supported clade (PP: 0.73; BS: 56) composed of two accessions from an adjacent population (*Chung 1805* & *3028*; 21 in Fig. 5). Similarly, *P. bipinnatifida* (6 in Fig. 5) is sister to an adjacent population (*Chung 1865*; 20 in Fig. 5) with strong support (PP: 1; BS: 100). The recently described *P. zhoui* (5 in Fig. 5) is sister to *Chung 1852* from Lipu (18 in Fig. 5) with moderate support values (PP: 0.86; BS: 63). Under the phylogenetic context, accessions of Clade 1 exhibit a strong geographic structure, with clade *P. jianghuaensis*-*P. cangwuensis* in the east (8 and 9 in Fig. 5), clade *P. zhoui*-*Chung 1852* in the southwest (5 and 18 in Fig. 5), and the remaining samples in the central region (6, 7, and 19–21 in Fig. 5).

**Fig. 6:**
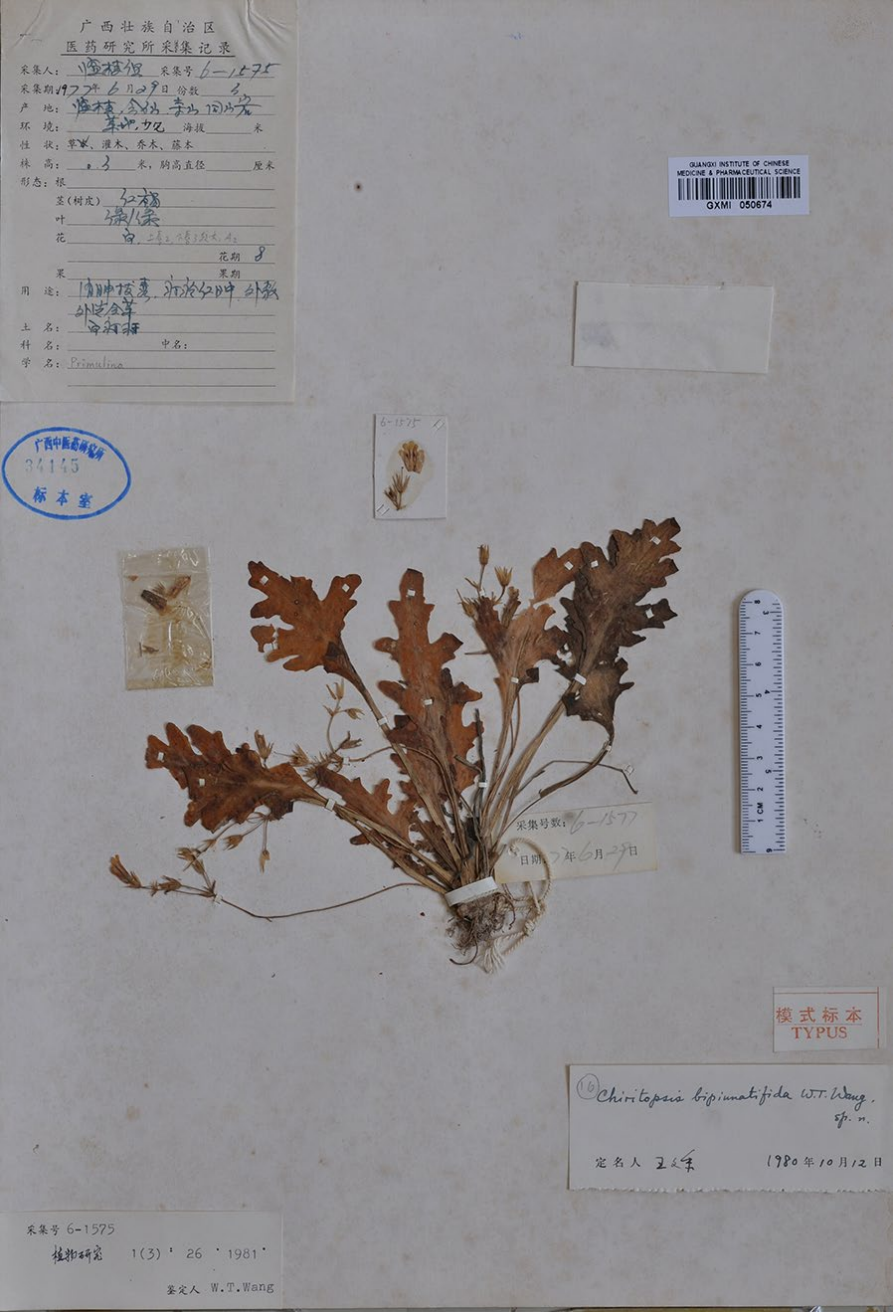
Holotype of *Chiritopsis bipinnatifida* W.T.Wang [*Lingui Exped. 6*-*1575* (GXMI)]

**Fig. 7:**
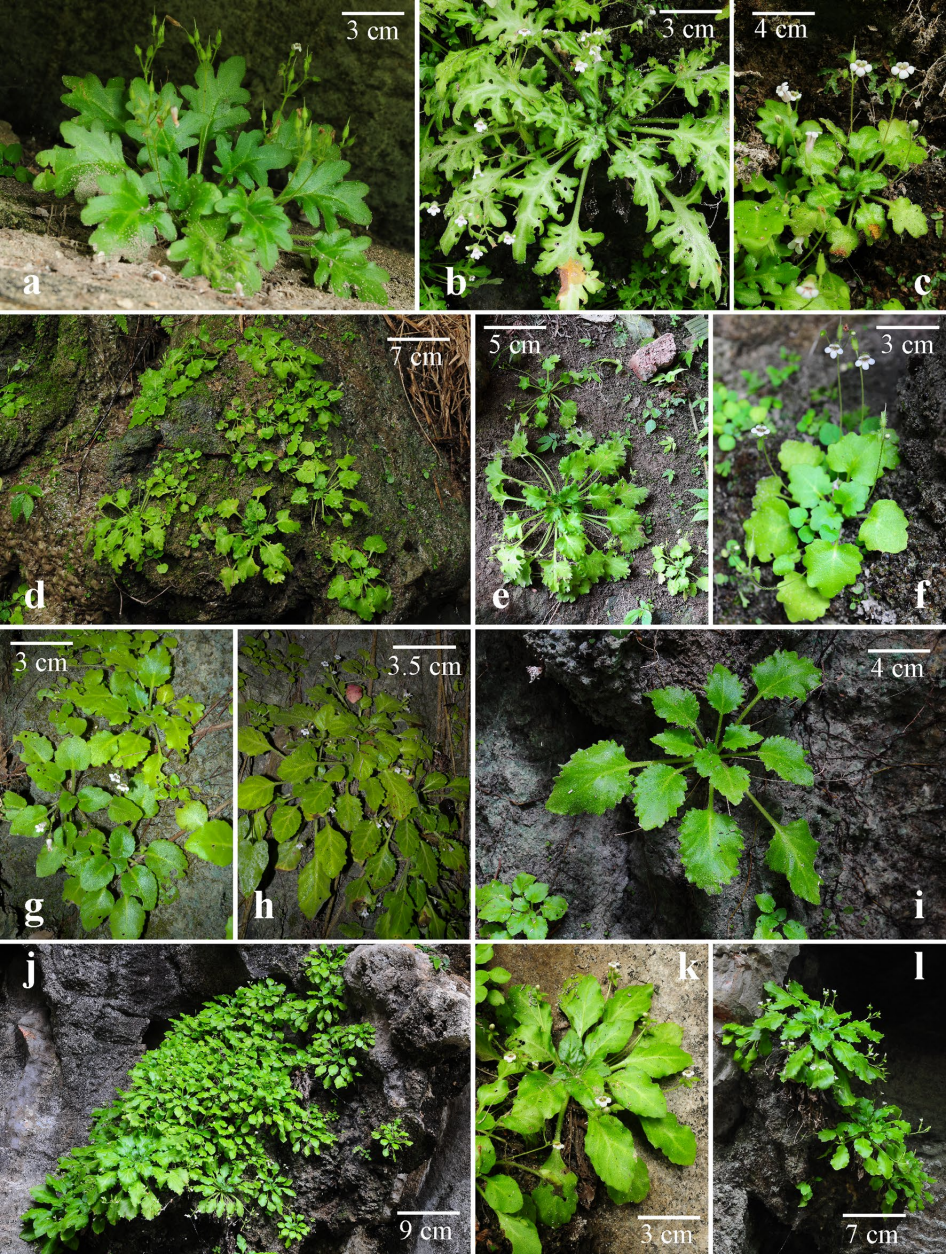
*Primulina bipinnatifida* (W.T.Wang) Yin Z.Wang & J.M.Li. **a**–**c** plants from type locality of *Chiritopsis bipinnatifida* W.T.Wang (*Chung 1863*); **d**–**f** plants from Linggui (*Chung 1865*); **g**–**i** plants from Yangshuo (*Chung 1858*); **j**–**l** plants from Lingchuan (*Chung 1805* & *3028*)

Within Clade 1, *Chiritopsis bipinnatifida* (≡ *P. bipinnatifida*) is the oldest name. However, the name “*bipinnatifida*” is somewhat misleading as the holotype (Fig. 6) is a single plant with pinnatifid leaves (i.e., “*pinnatim partitate*” in Wang 1981). In the type locality, we did observe plants with ‘bipinnatifid’ leaves (upper left in Figs. 3b and 7b); however, mature and flowering plants growing alongside with the ‘type’ morphology also possess leaves ranging from slightly sinuate, lobed, cleft, parted to bipinnatifid margins (Figs. 3b and 7a–c). In the type locality, the form with sinuate margins (Fig. 7c) are also present in its sister clade from the adjacent locality (*Chung 1865*) where plants with undulate to crispate margins grow alongside with the sinuate form (Fig. 7d–f). Indeed, within Clade 1, the form with sinuate to crispate margins that characterizing *P. jianghuaensis* (Fig. 3i–l) and *P. cangwuensis* (Fig. 3m–p) are commonly observed in most localities we sampled (Figs. 3 and 7), including Yangshuo (Fig. 7g–i; *Chung 1858*), the type locality of *P. lingchuanensis* (Fig. 3e–h) and *Chung 1805* & *3028* (Fig. 7j–l) that is phylogenetically the closest to *P. lingchuanensis* (Fig. 4a).

Geographically, plants in Clade 1 are distributed in northeastern Guangxi and adjacent Hunan, with a single outlier *P. zhoui* in central Guangxi (Fig. 5). Although we did not observe plants with leaves similar to *P. zhoui* in the type locality of *P. bipinnatifida*, considerable variation in leaf shapes also exists in *P. zhoui* with leaves shallowly to deeply cleft in the margins (Fig. 3r). Phylogenetically (Fig. 4a), *P. zhoui* is sister to a sample from Lipu (*Chung 1852*; Fig. 3s; 18 in Fig. 5) that is geographically the closest to *P. zhoui* (5 in Fig. 5) in our sampling. Interestingly, the population in Lipu possess reniform to ovate leaves with shallow pinnately cleft margins (Fig. 3s) very similar to some leaves of *P. zhoui* (Fig. 3r), while other leaves in the Lipu population are indistinguishable from those in *P. jianghuaensis* (Fig. 3j) and *P. cangwuensis* (Fig. 3n).

Judging from the fact that all five species discussed here were described based on individuals with extreme leaf forms and that the extent of variation in the respective type population had been neglected or ignore by previous authors (Wang 1981; Liu et al. 2006; Cai et al. 2014; Hong et al. 2018; Xin et al. 2018), our molecular data, as well as their identical floral morphology (Fig. 3) favor to recognize this clade as a single species distributed in northeastern Guangxi and southern Hunan (Fig. 5). The extent of morphological variation in its leaf shapes is likely resulted from random genetic drift in small and isolated population in the fragmented limestone karst landscape, as shown in other widespread species of *Primulina* (Gao et al. 2015; Wang et al. 2017) as well as other plant groups inhabiting caves and cave-like microhabitats of the Sino-Vietnamese limestone karsts (Chung et al. 2014; Tseng et al. 2019). Indeed, the taxonomical oversplitting in Clade 1 exemplifies Weber et al. (2011)’s observation that “*too many species have been described with numerous pairs or small groups of species growing in adjacent areas and differing only in slight, quantitative characters.*” Because *C. bipinnatifida* is the earliest valid name of the clade, *P. lingchuanensis*, *P. jianghuaensis*, and *P. cangwuensis* are proposed to be synonymized under *P. bipinnatifida*, with *P. zhoui* treated as a variety of *P. bipinnatifida* for its disjunct distribution and unique leaf shapes. In addition to their floral morphology, the recircumscribed *P. bipinnatifida* can be readily recognizable by the whitish variegation along the midveins and the secondary veins of the leaves, regardless its variable leaf shapes (Figs. 3 and 7).

### Clade 10

Clade 10 is composed of nine accessions (Fig. 4b), including three accessions sampled from the type localities (*P. repanda*, *P. cordifolia*, and *P. huangii*), as well as three additional accessions of *P. repanda* (*Chung 1815*, *1823*, and *Peng 22921*) and *P. cordifolia* (*Chung 1808*, *1826*, and *1828*) collected during our exploration in the limestone areas of Guangxi. Accessions in Clade 10 all have very similar corolla morphology (Fig. 2c, d, j, k, n, o).

**Fig. 8:**
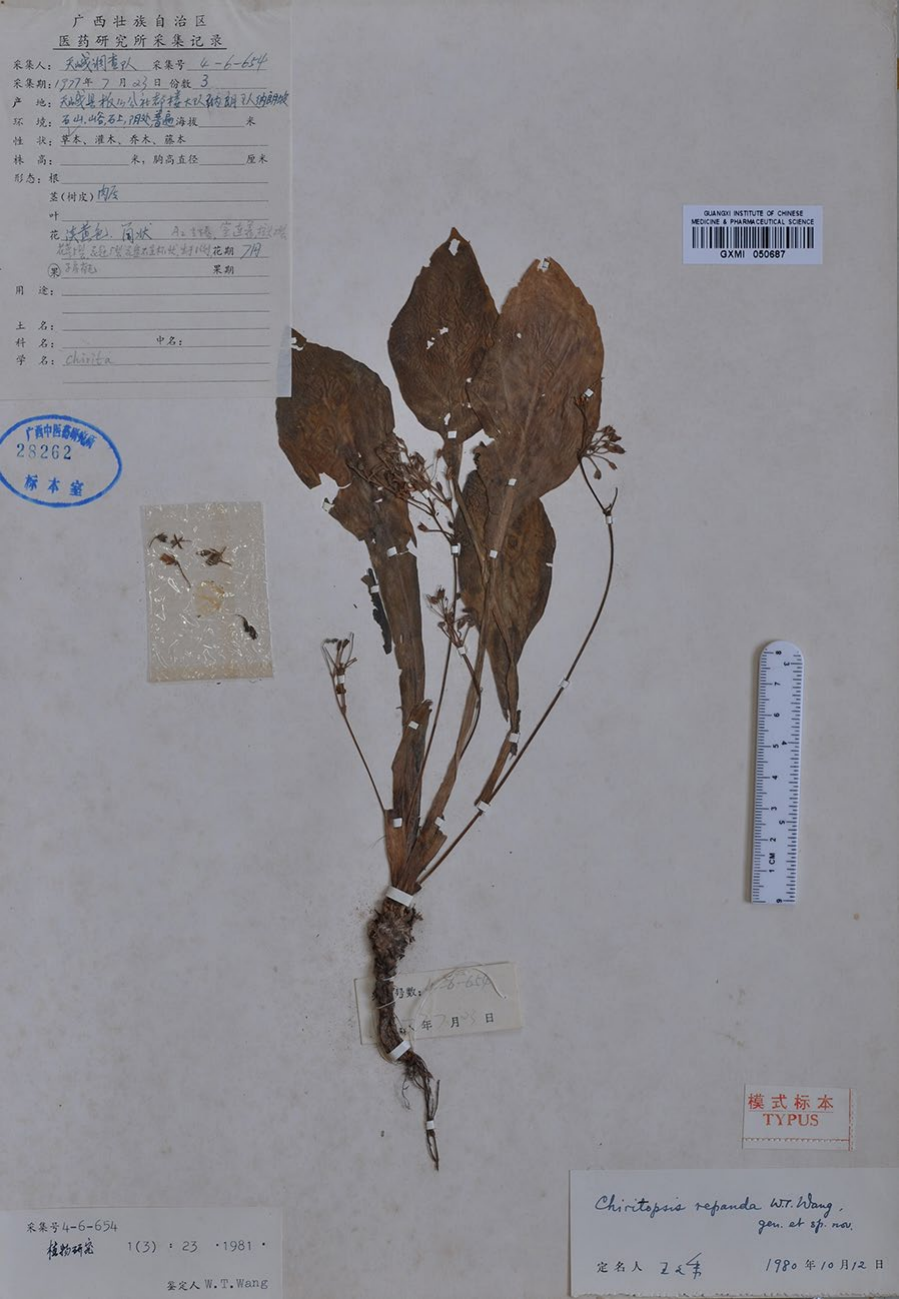
Holotype of *Chiritopsis repanda* W.T.Wang [*Tian’e Exped. 4*-*6*-*654* (GXMI)]

The holotype of *Chiritopsis repanda* (≡ *P. repanda*) is a plant possessing four petiolate leaves with slightly repand margins (Fig. 8). While one leaf is elliptic with attenuate and decurrent base extending through the petiole, three leaves are ovate with cuneate to oblique bases (Fig. 8). Our analyses (Fig. 4b) placed three additional accessions (*Chung 1815* and *1823*, and *Peng 22921*) in the same clade with *P. repanda* with strong support values (PP: 1; BS: 93). In general, these accessions possess leaves with entire to slightly sinuate leaf margins and attenuate and decurrent base extending through the petioles (Fig. 2e–g), though leaves with slightly cordate leaf bases could also be observed in those populations (e.g., Fig. 2b).

**Fig. 9:**
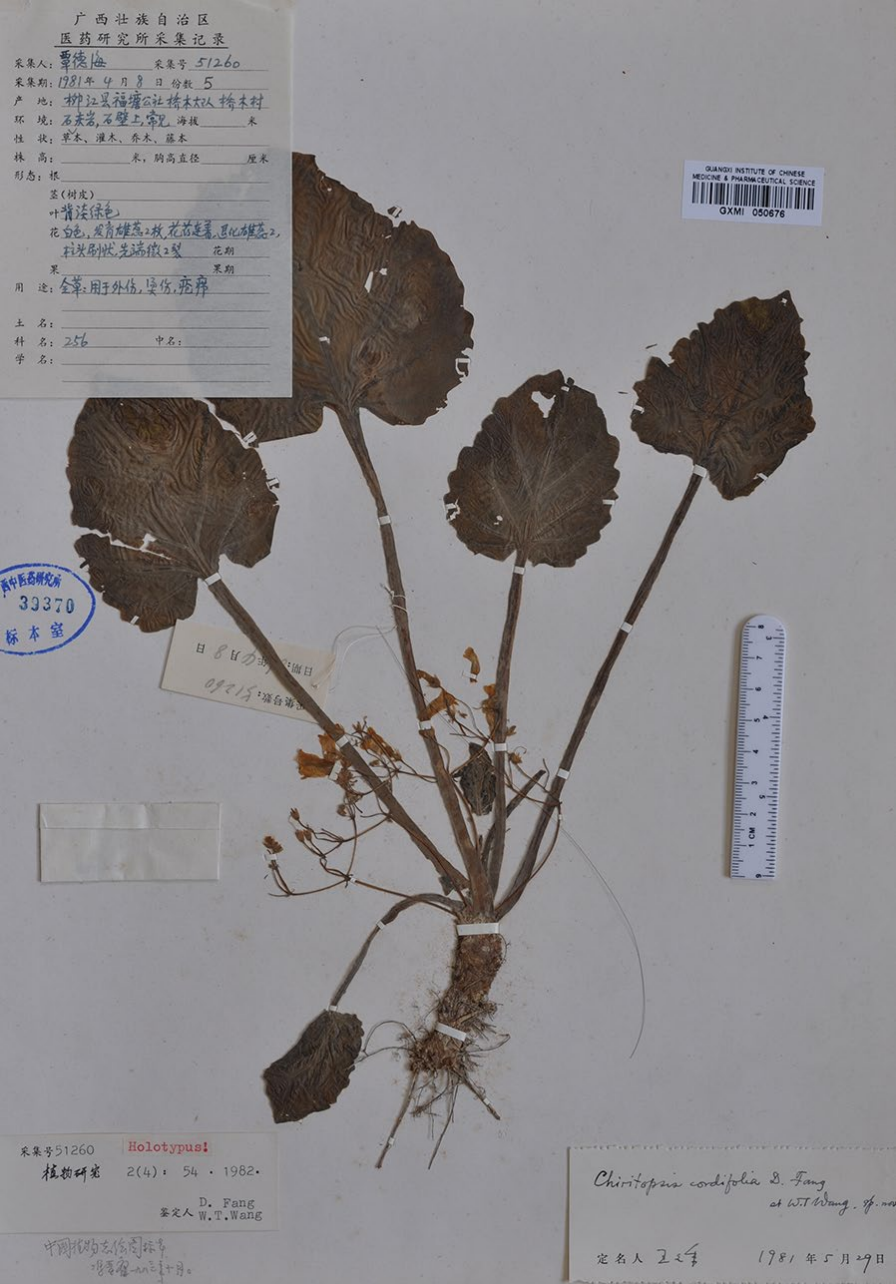
Holotype of *Chiritopsis cordifolia* D.Fang & W.T.Wang [*D.*-*H. Qin 51260* (GXMI)]

Within Clade 10 (Fig. 4b), three additional accessions of *P. cordifolia* (*Chung 1808*, *1826* and *1828*) form a moderately supported clade (PP: 098; BS: 67) sister to *P. cordifolia* from the type locality with strong support values (PP: 0.98; BS: 99). The holotype of *Chiritopsis cordifolia* (≡ *P. cordifolia*) is a plant with five petiolate leaves, all ovate with crenate margins and cordate to slightly truncate bases (Fig. 9). Those three additional accessions all possess leaves with cordate bases and slightly sinuate to crenate margins (Fig. 2p–r) that can be identified as *P. cordifolia*, though other character such as texture and hairiness are quite variable among these accessions.

The recently described *Primulina huangii* is (Fig. 2l–o) placed sister to the *P. cordifolia* clade with strong support values (PP: 1; BS: 96), though Xin et al. (2018) allied this unique species to *P. bipinnatifida* (Figs. 3 and 7) for their ‘bipinnatifid’ leaf margins. In the type locality of *P. huangii*, we also observed many plants possessed leaves varying from shallowly sinuate to deeply cleft margins (Fig. 2m), with many plants with cordate leaf based quite similar to *P. cordifolia*. The sister group relationship between *P. cordifolia* and *P. huangii* revealed by our data (Fig. 4b) suggests a likely evolutionary origin of their morphological similarity.

Recently, *P. niveolanosa* was described from Yizhou, central Guangxi that is morphologically comparable to *P. repanda* (Li et al. 2019). Although we have not seen this plant in the field, the overall morphology, especially the pubescence, of *P. niveolanosa* appears to be quite similar to three collections of *P. cordifolia*, *Chung 1817* (Fig. 2i), *Chung 1828* (Fig. 2p), and *Chung 1826* (Fig. 2r).

### Clade 3 and others

**Fig. 10:**
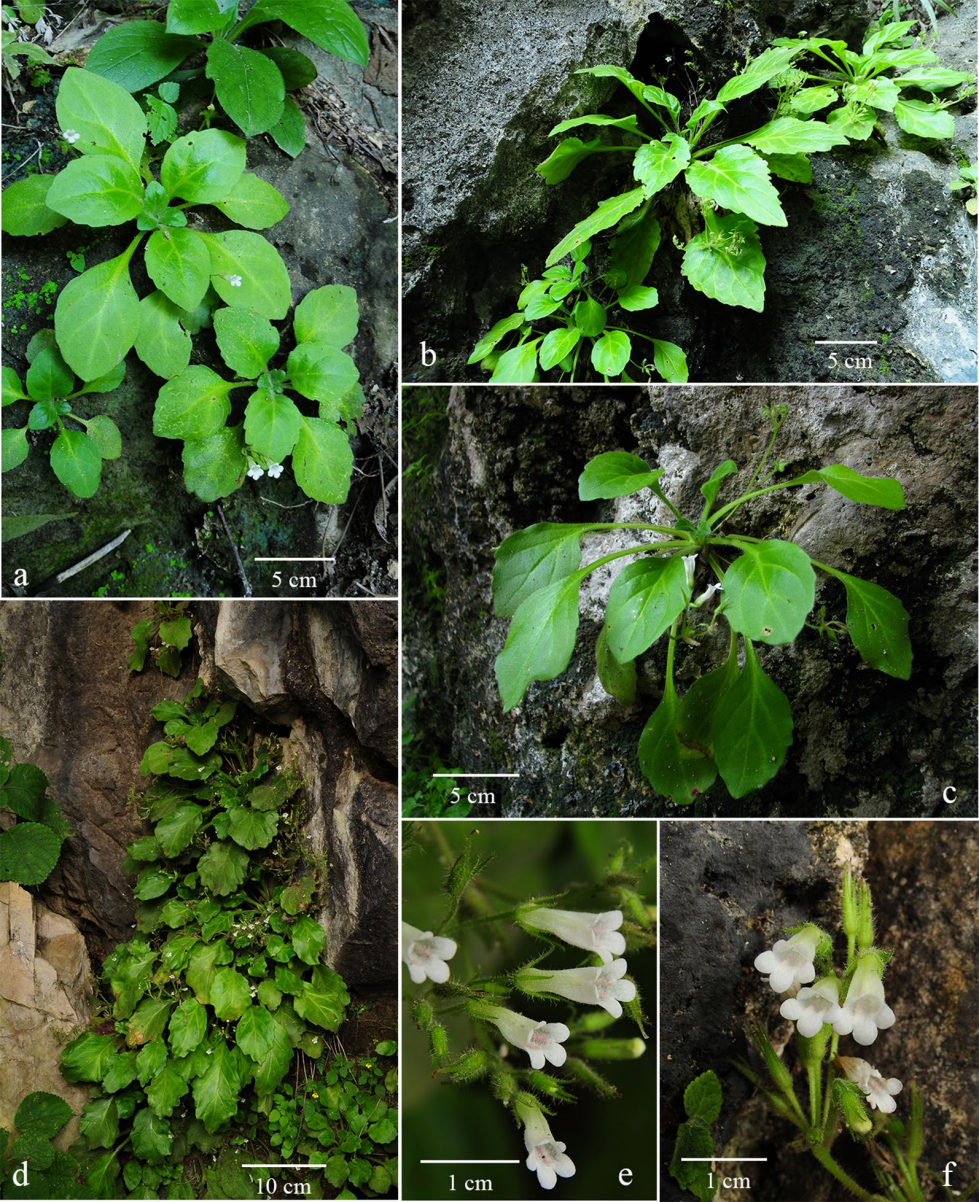
*Primulina subulata* var. *guilinensis* (W.T.Wang) W.B.Xu & K.F.Chung. **a**–**c** habit from Guilin; **d** habit from Hezhou (*K.*-*F. Chung 1845*); **e** flowers (side view); **f** flowers (face view). **a**–**c** from type locality of *Chiritopsis repanda* var. *guilinensis* (*K.*-*F. Chung 1806*)

Our phylogenetic analyses also necessitate the nomenclatural change for *Primulina repanda* var. *guilinensis* for its placement with *P. subulata* and *P. subulata* var. *yangchunensis* in Clade 3 (Fig. 4a), a relationship also evident in Kong et al. (2017). We propose to transfer it as *P. subulata* var. *guilinensis* comb. nov. (Fig. 10) to reflect its phylogenetic relationship and highlight its apparent morphological differences from *P. subulata* and *P. subulata* var. *yangchunensis*.

Congruent with the result of Kong et al. (2017), our analyses also indicate that *P. glandulosa* and *P. glandulosa* var. *yangshuoensis* are distantly related (Fig. 4a). However, raising the variety to the specific status is blocked by the existence of *P. yangshuoensis* Y.G.Wei & F.Wen (marked by red color in Fig. 4a). We therefore propose the new name *P. pseudoglandulosa* nom. nov. for this taxon. Kong et al. (2017) also showed the non-monophyly in *P. xiuningensis* among populations in Anhui, Zhejiang, and Hunan. Further study will be needed to clarify species circumscription in *P. xiuningensis*.

Our phylogenetic analyses further indicate that two unknown plants with Chiritopsis-like flowers are placed distantly from other species of the Chiritopsis-like *Primulina* (Fig. 4), supporting their recognition as new species. Here we provide detailed description for one species, *P. chingipengii*. Another species will be published by other research team shortly.

## Conclusions

Based on molecular phylogenetic analyses and field observations, we propose the following taxonomical changes, including one new species, one new name, two new combination, and three new synonyms.

### Taxonomic treatments

**Fig. 11:**
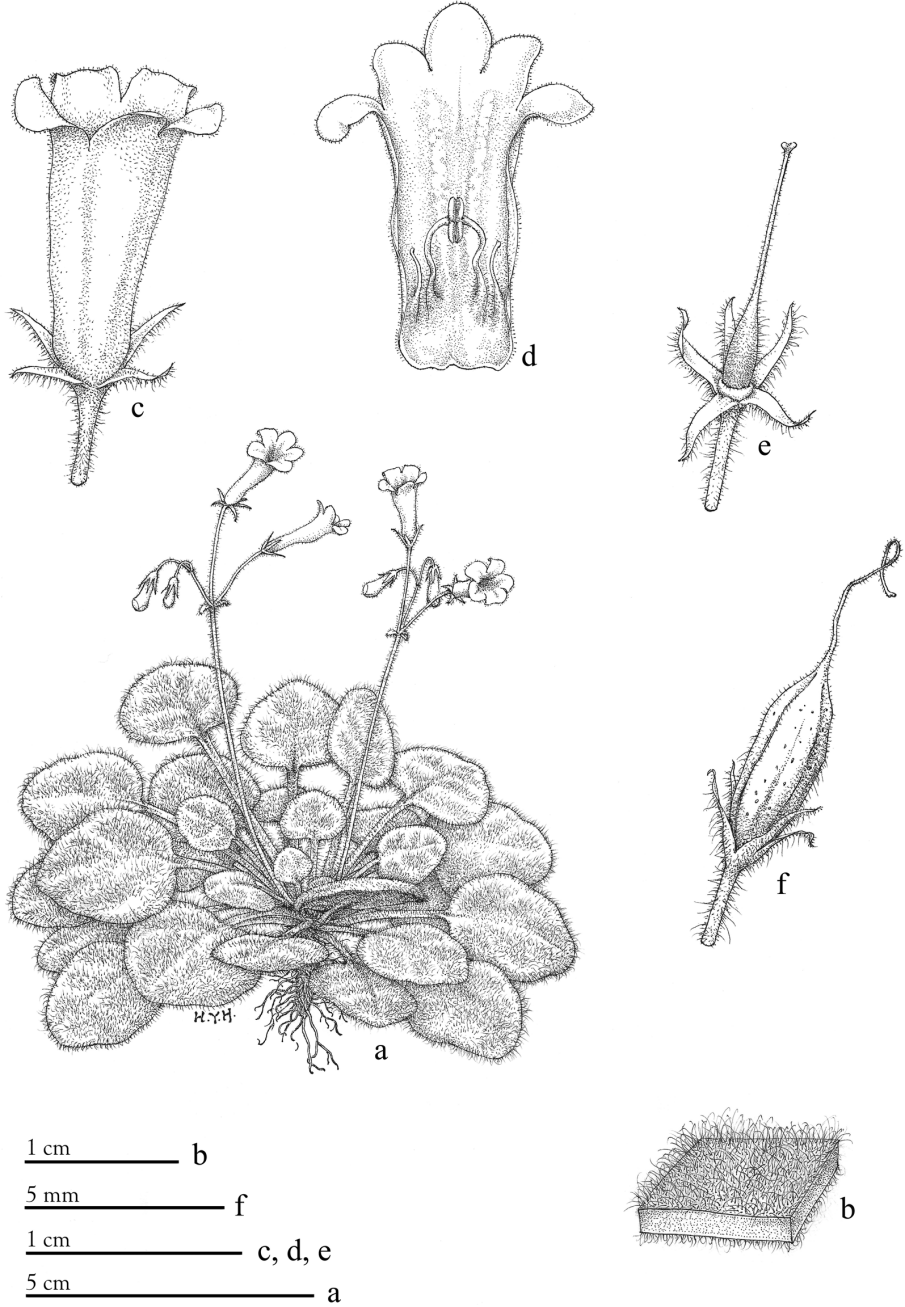
*Primulina chingipengii* W.B.Xu & K.F.Chung. **a** habit, **b** enlarged part of blade, **c** flower, **d** corolla opened showing stamens and staminodes, **e** calyx, pistil and disc, **f** capsule. (Drawn by Han-Yao Huang based on the holotype)

**Fig. 12:**
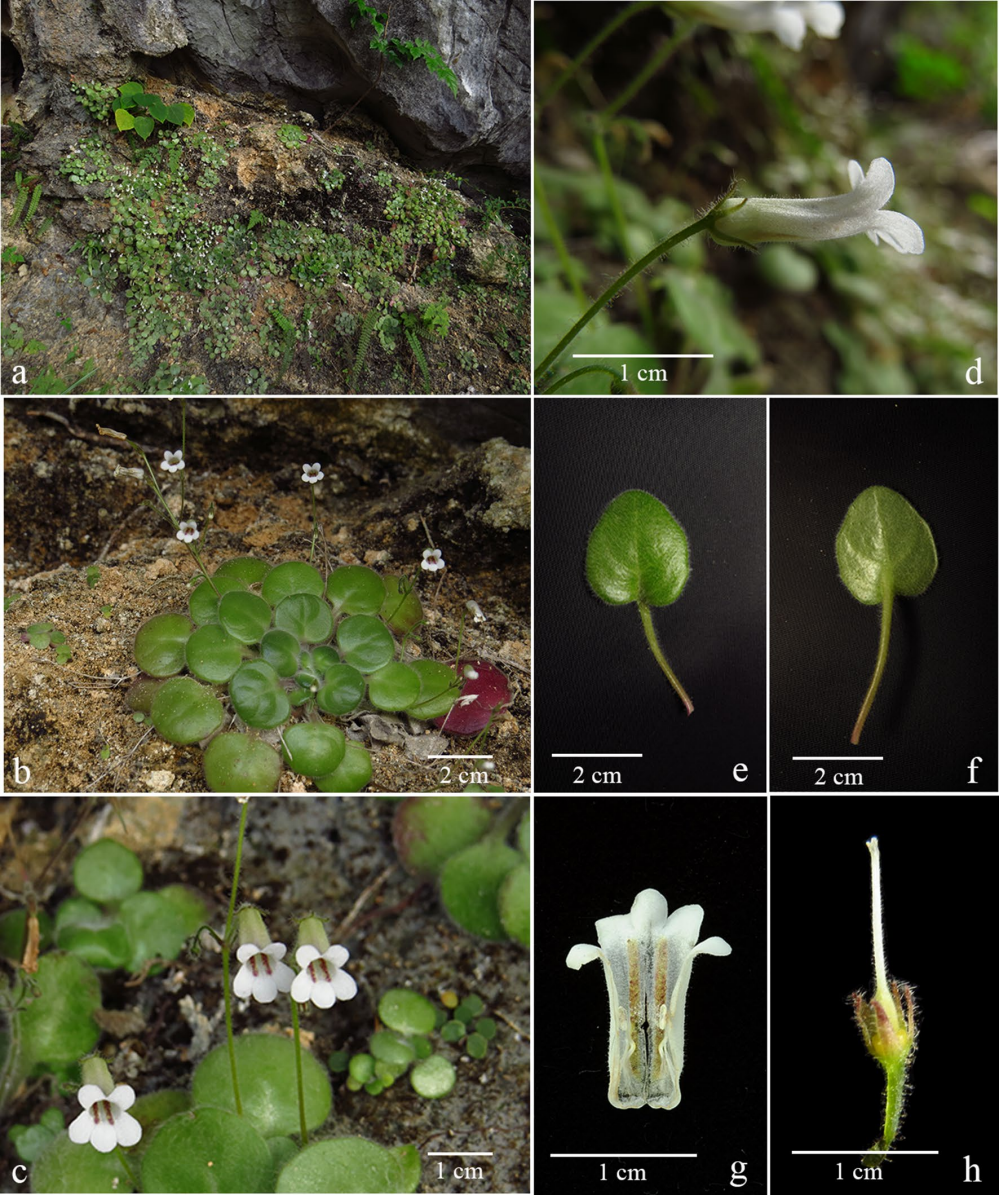
*Primulina chingipengii* W.B.Xu & K.F.Chung. **a** habitat, **b** habit, **c** flower (face view), **d** flower (side view), **e** upper surface of leaf, **f** lower surface of leaf, **g** opened corolla showing stamens and staminodes, **h** pistil and calyx. All taken from type locality (*W.*-*B. Xu* et al*. 13158*)

*Primulina chingipengii* W.B.Xu & K.F.Chung, sp. nov.: TYPE: CHINA, Guangxi, Du’an County, Chengjiang Town, Baxian Park, 23°54′59.11″N, 108°08′03.26″E, alt 235 m, 8 Oct 2016, *W.*-*B. Xu* et al*. 13158* (holotpye IBK, isotype HAST). 彭鏡毅小花苣苔 (Figs. 11 and 12).

Diagnosis: *Primulina chingipengii* is similar to *P. cordifolia* (Fig. 2h–k) in corolla shape, differing by the leaf blade carnose, broadly ovate, cordate to suborbicular, 2–3 × 1.2–2 cm, densely pubescent on both surfaces, base broadly cuneate, round to cordate in the former species.

Description: Herbs perennial. Rhizome subterete, 12–25 mm long, 4.5–9 mm wide. Leaves 8–23, basal, petiolate, carnose, papery when dry; petiole subterete, 2–6 cm long, 2–3 mm wide, densely pubescent; blades broadly ovate, cordate to suborbicular, 2–3 × 1.2–2 cm, densely pubescent on both surfaces, base broadly cuneate, round to cordate, margin repand, apex obtuse to rounded; lateral veins very inconspicuous, 2–3 on each side. Cymes 4–8, axillary, 1–3-branched, 4–15-flowered; peduncle 5-18 cm long, 1–2 mm in diam, spreadly pubescent and glandular-pubescent; bracts 2, opposite, linear-lanceolate, 3–4 × 0.5–0.8 mm, margin entire, pubescent and glandular-pubescent; pedicel 12–21 mm long, pubescent and glandular-puberscent. Calyx 5-parted to base, lobes lanceolate-linear, 3–4 × 0.5–1 mm, apex acuminate, outside pubescent and glandular-pubescent, inside sparsely puberulent, margins entire. Corolla white, 13–15 mm long, outside pubescent and glandular-pubescent, inside sparsely puberulent, with 2 pale purple stripes; corolla tube 10–12 mm long, 5–6 mm in diam. at the mouth, 3–4 mm in diam. at the base; limb distinctly 2-lipped; adaxial lip 2-parted to over the middle, lobes oblong, 2.5–4.5 × 2–3.5 mm; abaxial 3-lobed to over the middle, lobes oblong, 2.5–3.5 × 2.5–3 mm; stamens 2, adnate to 2.7 mm above the corolla tude base; filaments linear, 3.5–5 mm long, geniculate near the middle, sparsely puberulent; anthers 2–2.5 mm long, dorsifixed, glabrous; staminodes 2, 2–3 mm long, apex slightly capitate, glabrous, adnate to ca. 2.5 mm above the corolla tube base. Disc annular, 0.6–0.8 mm in height, margin slightly repand, glabrous. Pistil 10–13 mm long, ovary narrowly ovoid, 3.5–5 mm long, ca. 1.2 mm across, glandular-puberulent and puberulent; style 6–8 mm long, glandular-puberulent and puberulent; stigma obtrapeziform, ca. 0.5 mm long, 0.5–0.7 mm wide, apex 2-lobed. Capsule narrowly ellipsoidal, 6–8 mm long, 1.6–2.3 mm across, pubescent.

Distribution, habitat and ecology: *Primulina chingipengii* is only known from its type locality in Baxian Park, Chengjiang Town, Du’an County, Guangxi, China (Fig. 5). It grows on moist rock face at the entrance of a karst cave (Fig. 12a).

Phenology: Flowering from Sep to Oct, fruiting from Nov to Dec.

Etymology: The specific epithet honors Dr. Ching-I Peng (1950–2018), the late Research Fellow of Biodiversity Research Center, Academia Sinica, for his tremendous contribution to our knowledge of the East Asian flora and systematics of Asteraceae, *Ludwigia*, *Begonia*, and Sino-Vietnamese karst flora (Chung 2018).

Notes: *Primulina chingipengii* is similar to *P. cordifolia* in corolla shape, but it is easily distinguished from the latter by the leaf blades. Phenologically, *P. chingipengii* and *P. cordifolia* are also different. Our phylogenetic analyses revealed that *P. chingipengii* is placed in a strongly supported clade also including *P. albicalyx* B.Pan & LiH.Yang, *P. carinata* Y.G.Wei, F.Wen & H.Z.Lü, *P. fengshanensis* F.Wen & YueWang, and *P. pseudoeburnea* (D.Fang & W.T.Wang) Mich.Möller & A.Weber (Fig. 4b); however, only *P. chingipengii* possesses Chiritopsis-like corolla in this clade.

Additional specimens examined (paratypes): Guangxi, Du’an County, Chengjiang Town, Baxian Park, alt 235 m, 8 Oct 2016 *W.*-*B. Xu* et al*. 13157* (IBK), ibid., 13 Jul 2015, *K.*-*F. Chung* et al*. 2979* (HAST).

**Fig. 13:**
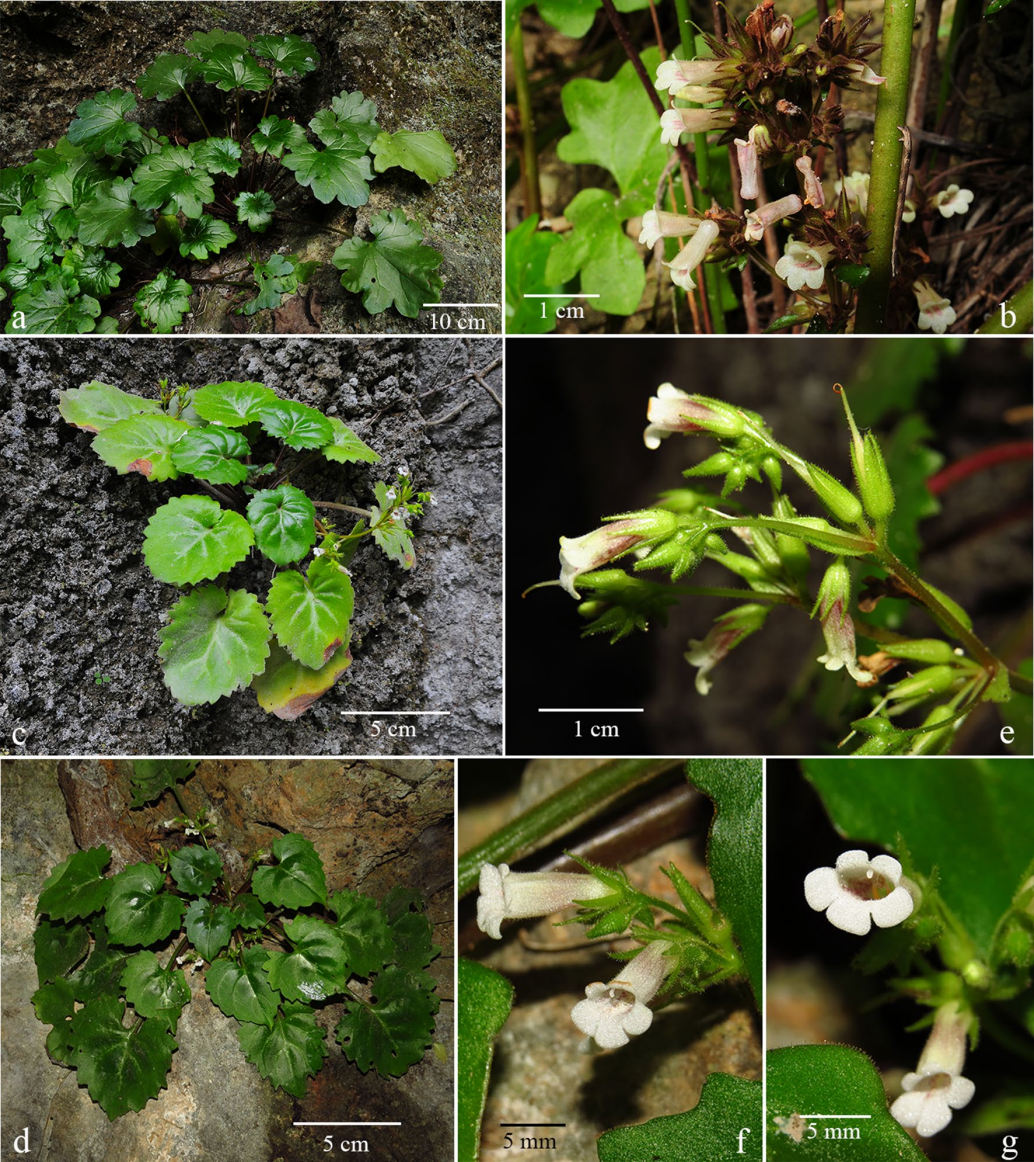
*Primulina glandulosa* (D.Fang, L.Zeng & D.H.Qin) Yin Z.Wang (**a**, **b**) and *Primulina pseudoglandulosa* W.B.Xu & K.F.Chung (**c**–**g**). **a** habit, **b** inflorescences; **c**, **d** habit, **e** inflorescences, **f** flowers (side view), **g** flowers (face view). **a**, **b** from type locality of *Chiritopsis glandulosa* D.Fang, L.Zeng & D.H.Qin [*Chung 1848* (HAST)]; **c**–**g** from type locality of *Chiritopsis glandulosa* var. *yangshuoensis* F.Wen, Y.Wang & Q.X.Zhang [*Chung 3022* (HAST)]

*Primulina pseudoglandulosa* W.B.Xu & K.F.Chung, nom. nov.: *Chiritopsis glandulosa* var. *yangshuoensis* F.Wen, YueWang & Q.X.Zhang in Guihaia 28(3): 291. 2008.—*Primulina glandulosa* var. *yangshuoensis* (F.Wen, YueWang & Q.X.Zhang) Mich.Möller & A.Weber in Taxon 60(3): 782. 2011 [non *Primulina yangshuoensis* Y.G.Wei & F.Wen in Taiwania 57(1): 56, *Fig.* *1 & 2A*. 2012.].—TYPE: CHINA, Guangxi, Yangshuo County, Yulonghe, alt 110–140 m, 24 Jun 2006, *F. Wen 06062401* (holotype BJFC, isotype IBK!). 陽朔小花苣苔 (Fig. 13c–g).

Description: Herbs perennial. Rhizome subterete, 3–7 cm long, 8–12 mm wide. Leaves 8–18, basal, petiolate, herbaceous, sometimes slightly carnose; petiole subterete, 4–8 cm long, 1.5–2.5 mm wide, sparsely pubescent; blades broadly ovate, cordate to suborbicular, 5.0–6.5 × 4.5–5.5 cm, sparsely pubescent on both surfaces, base cordate, margin dentate, apex acute to obtuse; subpinnipalmate, 2–3 on each side, sometimes white along the veins. Cymes 3–8, axillary, 1–3-branched, 8–15-flowered; peduncle 7–18 cm long, ca. 1.5 mm in diam, sparsely pubescent; bracts 2, opposite, lanceolate, 7–9 × 2–3 mm, margin entire, pubescent; pedicel 3–10 mm long, pubescent. Calyx 5-parted to base, lobes lanceolate-linear, 6–7 × 0.8–1 mm, apex acuminate, outside pubescent, inside sparsely puberulent, margins entire. Corolla white with pale purple, 10–13 mm long, outside sparsely pubescent, inside sparsely puberulent, with 2 pale purple stripes; corolla tube 6–10 mm long, 4–5 mm in diam. at the mouth, 3–4 mm in diam. at the base; limb distinctly 2-lipped; adaxial lip 2-parted to over the middle, lobes oblong, 1.5–2 × 2–3 mm; abaxial 3-lobed to over the middle, lobes oblong, 2–3 × 2.5–3.5 mm. Pistil 10–11 mm long, ovary narrowly ovoid, 3–4 mm long, ca. 1 mm across, sparsely puberulent; style 6–7 mm long, sparsely puberulent; stigma ca. 0.5–0.7 mm long, 0.5 mm wide. Capsule narrowly ellipsoidal, 7–8 mm long, 1–1.5 mm across, sparsely pubescent.

Distribution, habitat and ecology: *Primulina pseudoglandulosa* grows on moist shade cliffs of limestone, and also on moist rock face at the entrance of karst cave in Yangshuo, Guangxi (Fig. 5), at alt 120–150 m.

Phenology: Flowering from Jun to Jul, fruiting from Aug to Oct.

Notes: Because phylogenetic analyses show that *Primulina glandulosa* (Fig. 13a, b) and *P. glandulosa* var. *yangshuoensis* (Fig. 13c–g) are not closely related (Fig. 4a), it is inadequate to treat the latter a variety of the former. However, the existence of *Primulina yangshuoensis* Y.G.Wei & F.Wen blocks the direct transfer of *P. glandulosa* var. *yangshuoensis* to the specific status. The new name *Primulina pseudoglandulosa* (Fig. 13c–g) is proposed to highlight its morphological similarity to *P. glandulosa* (Fig. 13a, b).

Additional specimens examined: CHINA. Guangxi: Yangshuo County, Shenqiyan Cave, 5 Apr 2014, *M.*-*Q. Han & J. Guo G017* (IBK); Yulonghe, 16 Jul 2015, *K*-*.F. Chung* et al*. 3022* (HAST, IBK); Gaotian Town, 1 Sep 2010, *L. Wu & W.*-*B. Xu 10960* (IBK); Fuli Town, 28 Jul 2009, *W.*-*B. Xu* et al*. 09745* (IBK); ibid., 5 Sep 2012, *W.*-*B. Xu 11884* (IBK).

*Primulina subulata* var. *guilinensis* (W.T.Wang) W.B.Xu & K.F.Chung, comb. nov.: *Chiritopsis repanda* var. *guilinensis* W.T.Wang, Guihaia 12(4): 299. 1992.—*Primulina repanda var. guilinensis* (W.T.Wang) Mich.Möller & A.Weber in Taxon 60(3): 784. 2011.—TYPE: CHINA, Guangxi, Guilin City, Jiangjunqiao, 11 Aug 1960, *Y.*-*Y. Yang 6517* (holotype: PE!, isotype GXMI!). 桂林小花苣苔 (Fig. 10).

Description: Herbs perennial. Rhizome subterete, 2–8 cm long, 6–10 mm wide. Leaves 6–14, basal, petiolate, herbaceous; petiole flat, 2–10 cm long, 1.5–2.5 mm wide, pubescent; blades ovate, broadly ovate, elliptical, cordate, 4.0–12.5 × 2.5–6.5 cm, pubescent on both surfaces, base cuneate to cordate, margin repand to dentate, apex acute to obtuse; lateral veins 2–4 on each side. Cymes 2–6, axillary, 1–3-branched, 4–16-flowered; peduncle 3–16 cm long, ca. 1.5 mm in diam, pubescent; bracts 2, opposite, lanceolate, 6–8 × 2–2.5 mm, margin entire, pubescent; pedicel 4–21 mm long, pubescent. Calyx 5-parted to base, lobes lanceolate-linear, 5–7 × 0.8–1 mm, apex acuminate, outside pubescent, inside sparsely puberulent, margins entire. Corolla white, 8–14 mm long, outside sparsely pubescent, inside sparsely puberulent, with 2 vary pale purple stripes; corolla tube 6-10 mm long, 4.5–5.5 mm in diam. at the mouth, 3–4 mm in diam. at the base; limb distinctly 2-lipped; adaxial lip 2-parted to over the middle, lobes oblong, 1.5–2 × 2–3 mm; abaxial 3-lobed to over the middle, lobes oblong, 2.5–3.5 × 2.5–3.5 mm. Pistil 10-12 mm long, ovary narrowly ovoid, 3.5–4.5 mm long, ca. 1 mm across, puberulent; style 6–7 mm long, puberulent; stigma ca. 0.5–0.7 mm long, 0.5 mm wide. Capsule narrowly ellipsoidal, 5–6 mm long, 1–1.5 mm across, pubescent.

Distribution, habitat and ecology: *Primulina subulata* var. *guilinensis* grows on moist rock face at the entrance of karst cave, and also on moist shade cliffs of limestone, in Guangxi (Gongcheng, Guilin, Hezhou, Pingle, Quanzhou, Zhongshan) and Hunan (Jiangyong), at alt 100–230 m. The record of *Chiritopsis repanda* var. *guilinensis* in Luzhai County, Liuzhou City in Li and Wang (2004) is incorrect based on our field observation.

Phenology: Flowering from Jul to Aug, fruiting from Sep to Oct.

Notes: Phylogenetic analyses indicate that *Primulina repanda* var. *guilinensis* is placed in a strongly supported clade that also includes *P. subulata* and *P. subulata* var. *yangchunensis*, distantly related to *P. repanda* (Fig. 4). To better reflect its phylogenetic position, we propose to make the current nomenclatural change.

Additional specimens examined: CHINA. Guangxi: Guilin City, Qixing District, 18 Jul 2009, *W.*-*B. Xu* et al*. 09774* (IBK, HAST). Quanzhou County, Shitang Town, 1 Aug 2014, *Quanzhou Exped. 450324140801022* (IBK, GXMG). Gongcheng County, Xiling Town, 28 Aug 2006, *W.*-*B. Xu & Y.*-*Y. Liang 06037* (IBK). Hezhou City, Babu District, 4 Oct 2008, *W.*-*B. Xu & W.*-*H. Wu 08212* (IBK), ibid., 26 Jul 2009, *W.*-*B. Xu* et al*. 09788* (IBK, HAST); Etang Town, 10 Jul 2009, *Y. Liu & W.*-*B. Xu 091740* (IBK), ibid., 7 Aug 2012, *W.*-*B. Xu* et al*. 11714* (IBK). Pingle County, Yuantou Town, 4 Apr 2014, *M.*-*Q. Han & J. Guo G010* (IBK). Zhongshan County, Qingtang Town, 4 Apr 2014, *M.*-*Q. Han & J. Guo G009* (IBK). Hunan: Jiangyong County, Lanxi Town, 25 Jun 2011, *X.*-*L. Yu 110611* (CSFI); ibid., 9 Apr 2018, *C.*-*R. Lin* et al*. YY190* (IBK).

*Primulina bipinnatifida* (W.T.Wang) Yin Z.Wang & J.M.Li in J. Syst. Evol. 49(1): 60. 2011.—*Chiritopsis bipinnatifida* W.T.Wang, Bull. Bot. Res., Harbin 1(3): 26. 26–28, *pl. 1(9*–*11), pl. 4(1)*. 1981.—TYPE: CHINA, Guangxi, Lingui County, Huixian Town, Sishan, 29 Jun 1977, *Lingui Exped. 6*-*1575* (holotype: GXMI!). 羽裂小花苣苔 (Figs. 3, 6, 7).

*Chiritopsis lingchuanensis* Yan Liu & Y.G.Wei in Acta Phytotax. Sin. 44(3): 340–343, *Fig.* *1*. 2006.—*Primulina lingchuanensis* (Yan Liu & Y.G.Wei) Mich.Möller & A.Weber in Taxon 60(3): 783. 2011.—TYPE: CHINA, Guangxi, Lingchuan County, Dajing Town, alt 340 m, 11 Sep 2004, *Y. Liu L1085* (holotype: IBK!, isotypes: IBK!, PE), syn. nov. (Fig. 3e–h).

*Primulina jianghuaensis* K.M.Liu & X.Z.Cai in Nord. J. Bot. 32(1): 70–73, *Fig.* *1 & 2*. 2014.—TYPE: CHINA, Hunan Province, Jianghua County, 24°51′21.05″N, 111°42′58.22″E, at 397 m, 23 Jul 2012, *K.M. Liu & X.Z. Cai 31269* (holotype: HNNU, isotypes: HNNU), syn. nov. (Fig. 3i–l).

*Primulina cangwuensis* X.Hong & F.Wen in Ann. Bot. Fennici 55(1–3): 38, *Fig.* *1 & 2*. 2018.—TYPE: CHINA, Guangxi, Wuzhou City, Cangwu County, 23°50′N, 111°32′E, alt 200 m, 27 Sep 2010, *HX 100907* (holotype: IBK!, isotype: ANU), syn. nov. (Fig. 3m–p).

Distribution: *Primulina bipinnatifida* is distributed in northeastern Guangxi (Cangwu, Guilin, Hezhou, Lingchuan, Lipu, Pingle, Xing’an, Yangshuo) and adjacent areas of Hunan Province (Jianghua) (Fig. 5).

Notes: Our field observations (Fig. 3, 7) and phylogenetic analyses (Fig. 4a) support to recognize *P. bipinnatifida* a morphologically variable species distributed in northeastern Guangxi and southern Hunan (Fig. 5). Despite its variable leaf shapes, the recircumscribed *P. bipinnatifida* is readily recognizable by the white variegation along the midveins and the secondary veins of the leaves (Fig. 3, 7). Population genetic and phylogeographic studies of *P. bipinnatifida* are currently underway to understand evolutionary mechanisms underlying this variable species.

Additional specimens examined: CHINA. Guangxi: Guilin City, Yanshan Town, 10 Oct 1950, *J.X. Zhong 808581* (IBK). Lingui County, Huixian Town, Aug 2005, *W.*-*B. Xu LG*-*001* (IBK); ibid., 1 May 2009, *W.*-*B. Xu & Y. Liu 09409* (IBK), ibid., 28 Jul 2009, *W.*-*B. Xu* et al*. 09741 & 09742* (IBK), ibid., 14 Jul 2015, *K.*-*F. Chung* et al*. 2987* (HAST, IBK). Hezhou City, Kaishan Town, 8 Jul 2015, *K.*-*F. Chung* et al*. 2922* (HAST, IBK). Lingchuan County, Dajing Town, 30 Aug 2006, *W.*-*B. Xu 06039* (IBK), ibid., 6 Jul 2007, *B. Pan & W.*-*B. Xu 07240* (IBK), ibid., 17 Jul 2015, *K.*-*F. Chung* et al*. 3028* (HAST, IBK); Chaotian Town, 18 Jul 2009, W.-*B. Xu* et al*. 09773* (IBK, HAST), ibid., 15 Jul 2015, *K.*-*F. Chung* et al*. 2999* (HAST, IBK). Pingle County, Qiaoting Town, 27 Jul 2017, *Pingle Exped. 450330170727076LY* (IBK, GXMG); Tong’an Town, 9 Nov 2018, *Pingle Exped. 450330181109001LY* (IBK, GXMG). Yangshuo County, Putao Town, 1 Aug 2006, *W.*-*B. Xu 06038* (IBK); Xingping Town, 28 Jul 2009, *W.*-*B. Xu* et al*. 09797* (IBK, HAST), ibid., 16 Jul 2015, *K.*-*F. Chung* et al*. 3013* (HAST, IBK); Baisha Town, 28 Jul 2009, *W.*-*B. Xu* et al*. 09744* (IBK). Lipu County, Qingshan Town, 25 Aug 2006, *W.*-*B. Xu & Y.*-*Y. Liang 06035* (IBK), ibid., 27 Jul 2009, *W.*-*B. Xu* et al*. 09753* (IBK, HAST). Cangwu County, Shiqiao Town, 10 Jul 2009, *Y. Liu & W.*-*B. Xu 091743 & 091744* (IBK); ibid., 26 Jul 2009, *W.*-*B. Xu* et al*. 09789* (IBK, HAST). Xing’an County, Baishi Town, 15 Jul 2015, *K.*-*F. Chung* et al*. 2998* (HAST, IBK). Hunan: Jianghua, Daxu Town, 8 Jul 2015, *K.*-*F. Chung* et al*. 2925* (HAST, IBK); 9 Jul 2015, *K.*-*F. Chung* et al*. 2932* (HAST, IBK).

*Primulina bipinnatifida* var. *zhoui* (F.Wen & Z.B.Xin) W.B.Xu & K.F.Chung, comb. & stat. nov.: *Primulina zhoui* F.Wen & Z.B.Xin in Taiwania 63(1): 54–56, *fig.* *1 & 2.* 2018—TYPE: CHINA, Guangxi, Liuzhou City, Liujiang District, Liyong Town, 24°13′N, 109°28′E, Alt. 117 m, 18 Jul. 2015, *F. Wen* et al*. WF150718*-*01* (holotype IBK!; isotypes IBK, KUN, PE, TAI!). 周氏小花苣苔 (Fig. 3q, r).

Notes: Because of the disjunct distribution in central Guangxi and its unique leaf shape, *Primulina zhoui* is treated as a variety under *P. bipinnatifida*.

## Additional file

**Additional file 1.** Taxon: NCBI accession numbers (ITS/trnL-F/psbA-trnH), and voucher information [Geography, Collector number (herbarium)] of newly generated DNA sequences or reference.

## Data Availability

All DNA sequences generated in this study have been registered to GenBank.
