# Supplementary material for: Molecular systematics of Chiritopsis-like Primulina (Gesneriaceae): one new species, one new name, two new combinations, and new synonyms
Source: Bot Stud. 2019 Aug 29;60:18. doi: 10.1186/s40529-019-0266-x (PMC6715764; doi:10.1186/s40529-019-0266-x)
Supplement: Supplementary file 1 — Additional file 1. Taxon: NCBI accession numbers (ITS/trnL-F/psbA-trnH), and voucher information [Geography, Collector number (herbarium)] of newly generated DNA sequences or reference. [file 40529_2019_266_MOESM1_ESM.docx]

**Additional File 1.** *Taxon*: NCBI accession numbers (ITS/*trnL-F*/*psbA-trnH*), and voucher information [Geography, *Collector number* (herbarium)] of newly generated DNA sequences or reference. Vouchers marked by an asterisk sign (*) indicate specimens of Chiritopsis-like *Primulina* collected in type locality.

*Petrocodon hancei* (Hemsl.) A.Weber & Mich.Möller, KY796057/KY796059/KY796061, China, Guangdong, Shaoguan, Renhua, Danxiashan National Park, *C.-I Peng 22903* (HAST); *Pe. dealbatus* Hance, JX506959/JX506767/JX507075, China, Guangxi, *K.-F. Chung 1872* (HAST); *Primulina albicalyx* B.Pan & LiH.Yang, KY395016/ KY393613/-- (Kong et al. 2017); *Pr. alutacea* F.Wen, B.Pan & B.M.Wang, KY394847/ KY394847/-- (Kong et al. 2017); *Pr. argentea* XinHong, F.Wen & S.B.Zhou, KY394848/KY393442/-- (Kong et al. 2017); *Pr. baishouensis* (Y.G.Wei, H.Q.Wen & S.H.Zhong) Y.Z.Wang, JX506853/JX506742/JX506962, China, Guangxi, Yungfu, Baishuoyan, *K.-F. Chung 1876* (HAST); *Pr. beiliuensis* B.Pan & S.X.Huang, JX506882/JX506774/JX506970, China, Guangxi, Beiliu, *B. Pan 012* (IBK); *Pr. beiliuensis* var. *fimbribracteata* F.Wen & B.D.Lai, KY394851/KY393445/-- (Kong et al. 2017); *Pr. bicolor* (W.T.Wang) Mich.Möller & A.Weber, KY394852/KY393446/-- (Kong et al. 2017); *Pr. bipinnatifida* (W.T.Wang) Y.Z.Wang & J.M.Li, JX506854/JX506743/JX506964, China, Guangxi, Lipu, Qingshan, Shuangjiang, *K.-F. Chung 1852* (HAST), JX506855/JX506744/JX506965, China, Guangxi, Yangshuo, Xingping, Yucun/Dahebei, *K.-F. Chung 1858* (HAST), JX506878/JX506770/JX506966, China, Guangxi, Linggui, Huixiang, Sishan, *K.-F. Chung 1863* (HAST)*, JX506879/JX506771/JX506967, Linggui, Huixiang, Baoshan, *K.-F. Chung 1865* (HAST), JX506862/JX506751/JX507014, Lingchuan, Chaotian, Nanxu, *K.-F. Chung 1805* (HAST), MK503716/--/--, *K.-F. Chung 3028* (HAST); *Pr. bobaiensis* Q.K.Li, Q.Zhang & W.L.Li, JX506880/JX506772/JX506968, China, Guangxi, Bobai, Wangmo, *W.-B. Xu et al. 091775* (IBK); *Pr. brachytricha* (W.T.Wang & D.Y.Chen) R.B.Mao et Y.Z.Wang, KY394856/ KY393450/-- (Kong et al. 2017); *Pr. bullata* S.N.Lu & F.Wen, KY394857/KY393451/-- (Kong et al. 2017); *Pr. cangwunesis* X.Hong & F.Wen (≡ *Pr. bipinnatifida*), JX506877/JX506769/JX506963, China, Guangxi, Cangwu, Shiqiao, *K.-F. Chung 1842* (HAST); *Pr. cardaminifolia* YanLiu & W.B.Xu, JX506739/JX506740/-- (Xu et al. 2013); *Pr. carinata* Y.G.Wei, F.Wen & H.Z.Lü, KY394858/KY393452/-- (Kong et al. 2017); *Pr. chingipengii* W.B.Xu & K.F.Chung, sp. nov., MH032852/MH032843/MH032844, China, Guangxi, Duan, Chengjiang, *K.-F. Chung 2979* (HAST)*; *Pr. chizhouensis* XinHong, S.B.Zhou & F.Wen, KY394861/KY393455/-- (Kong et al. 2017); *Pr. confertiflora* (W.T.Wang) Mich.Möller & A.Weber, KC190195/KC190202/KC190209, China, Guangdong, Yangshan, *W.-B. Xu 11778* (IBK)*; *Pr. cordata* Mich.Möller & A.Weber, KC190200/KC190207/KC190214, China, Guangxi, Yangshuo, *H.-Y. Huang 10* (HAST); *Pr. cordifolia* (D.Fang & W.T.Wang) Y.Z.Wang, JX506884/JX506776/JX506975, China Guangxi, Bama, Baimuodong, *K.-F. Chung 1828* (HAST), JX506858/JX506747/JX506974, Donglan, Wuzhuang, Shangxu, *K.-F. Chung 1826* (HAST), JX506856/JX506745/JX506972, China, Guangxi, Rongan, Siding, Sanpo, *K.-F. Chung 1808* (HAST), JX506857/JX506746/JX506973, Liujiang, Luoman, Futang, *K.-F. Chung 1817* (HAST)*; *Pr. crassirhizoma* F.Wen, BoZhao & XinHong, KY394864/KY393458/-- (Kong et al. 2017); *Pr. cruciformis* (Chun) Mich.Möller & A.Weber, KY394986/KY393617/-- (Kong et al. 2017); *Pr. danxiaensis* (W.B.Liao, S.S.Lin & R.J.Shen) W.B.Liao & K.F.Chung, JX506886/JX506778/JX506977, China, Guangdong, Shaoguan, Renhua, Danxiashan National Park, *C.-I Peng 22865* (HAST)*; *Pr. davidioides* F.Wen & XinHong, KY394999/KY393629/-- (Kong et al. 2017); *Pr. debaoensis* NengJiang & HongLi, KY394868/KY393462/-- (Kong et al. 2017); *Pr. depressa* (Hook.f.) Mich.Möller & A.Weber, KY786295/KY786303/KY786315, China, Guangdong, Shaoguan, Qujiang, *C.-I Peng 24001* (HAST); *Pr. dichroantha* F.Wen, Y.G.Wei & S.B.Zhou, MK369971/MK369986/MK370002, China, Guangxi, *H.-T. Wu & O.-W. Wang 14051001* (IBK); *Pr. diffusa* XinHong, F.Wen & S.B.Zhou, KY394871/KY393465/-- (Kong et al. 2017); *Pr. dongguanica* F.Wen, Y.G.Wei & R.Q.Luo, KY394872/ KF498226/-- (Kong et al. 2017); *Pr. drakei* (B.L.Burtt) Mich.Möller & A.Weber, KY394873/KY393467/-- (Kong et al. 2017); *Pr. dryas* (Dunn) Mich.Möller & A.Weber, KY394875/KY393469/-- (Kong et al. 2017); *Pr. duanensis* F.Wen & S.L.Huang, KY394877/KY393471/-- (Kong et al. 2017); *Pr. eburnea* (Hance) Y.Z.Wang, JX506891/JX506783/JX506984, China, Guangxi, Liujiang, Baipeng, Fenglong, *C.-I Peng 22908* (HAST), KY786299/KY786305/KY786317, China, Guangdong, Shaoguan, Qujiang, Maba, *C.-I Peng 24012* (HAST); *Pr. effusa* F.Wen & B.Pan, MK369976/MK369991/MK370003, China, Guangdong, *B. Pan et al. P468* (IBK)*; *Pr. fangdingii* ined., JX506956/JX506847/JX507071, *W.-B. Xu et al. 12957* (IBK); *Pr. fengkaiensis* Z.L.Ning & M.Kang, MK369975/MK369990/MK370004, China, Guaongdong, *M. Kang et al. GDFK06* (IBSC); *Pr. fengshanensis* F.Wen & YueWang, MK369970/MK369985/MK370005, China, Guangxi, Fengshan, *F. Wen 06100101* (IBK); *Pr. fimbrisepala* (Hand.-Mazz.) Y.Z.Wang, JX506894/JX506786/JX506989, China, Guangdong, Shaoguan, Renhua, Danxiashan National Park, *C.-I Peng 22863* (HAST); *Pr. fimbrisepala* var. *mollis* (W.T.Wang) Mich.Möller & A.Weber, JX506895/JX506787/JX506990, China, Guangxi, *B. Pan s.n.* (IBK); *Pr. flavimaculata* (W.T.Wang) Mich.Möller & A.Weber, MK369974/MK369989/MK370006, China, Hainan, *K.-F. Chung 3988* (HAST); *Pr. floribunda* (W.T.Wang) Mich.Möller & A.Weber, KY394886/ KY393480/-- (Kong et al. 2017); *Pr. fordii* (Hemsl.) Y.Z.Wang, MK369981/MK369996/MK370007, China, Guangxi, *K.-F. Chung 4198* (HAST); *Pr. gemella* (D.Wood) Y.Z.Wang, FJ501345/FJ501523/-- (Möller et al. 2009); *Pr. gigantea* F.Wen, B.Pan & W.H.Luo, KY394984/ KY393615/-- (Kong et al. 2017); *Pr. glandulosa* (D.Fang, L.Zeng & D.H.Qin) Y.Z.Wang, JX506897/JX506789/JX506993, China, Guangxi, Pingle, Qinglong, Longshan, *K.-F. Chung 1848* (HAST)*; *Pr. glandulosa* var. *yangshuoensis* (F.Wen, YueWang & Q.X.Zhang) Mich.Möller & A.Weber (≡ *Pr. pseudoglandulosa* W.B.Xu & K.F.Chung, nom. nov.), JX506898/JX506790/JX506994, China, Guangxi, Yangshuo, Yulonghe, *K.-F. Chung 1859* (HAST)*; *Pr. gongchengensis* Y.S.Huang & YanLiu, KY394889/KY393483/-- (Kong et al. 2017); *Pr. gueilinensis* (W.T.Wang) Y.Z.Wang & YanLiu, JX506860/JX506749/JX506996, China, *M.-Q. Han et al. G016* (IBK); *Pr. guigangensis* L.Wu & Q.Zhang, KY394892/KY393486/-- (Kong et al. 2017); *Pr. guihaiensis* (Y.G.Wei, B.Pan & W.X.Tang) Mich.Möller & A.Weber, JX506900/JX506792/JX506997, China, Guangxi, Lingchuang, Dajing, *K.-F. Chung 1801* (HAST); *Pr. guizhongensis* BoZhao, B.Pan & F.Wen, JX506902/JX506794/JX506999, China, Guangxi, Laibin, *W.-B. Xu et al. 08010* (IBK); *Pr. halongensis* (Kiew & T.H.Nguyên) Mich.Möller & A.Weber, KY394895/ KY393489/-- (Kong et al. 2017); *Pr. hedyotidea* (Chun) Y.Z.Wang, JX506905/JX506797/JX507000, China, Guangxi, Longzhou, *W.-B. Xu et al. 12541* (IBK); *Pr. heterochroa* F.Wen & B.D.Lai, KY394898/KY393492/-- (Kong et al. 2017); *Pr. heterotricha* (Merr.) Y.Dong & Y.Z.Wang, KY394899/KY393493/-- (Kong et al. 2017); *Pr. hezhouensis* (W.H.Wu & W.B.Xu) W.B.Xu & K.F.Chung, JX506906/JX506798/JX507003, China, Guangxi, Hezhou, Etang, Lugang, *K.-F. Chung 2914* (HAST)*; *Pr. hochiensis* (C.C.Huang & X.X.Chen) Mich.Möller & A.Weber, JX506903/JX506795/JX507001, China, Guangxi, Luocheng, *Y. Liu L1231* (IBK); *Pr. huaijiensis* Z.L.Ning & J.Wang, KY394901/KY393495/-- (Kong et al. 2017); *Pr. hunanensis* K.M.Liu & X.Z.Cai, MH032853/MH032842/MH032845, China, Hunan, Jianghua, Daxu, *K.-F. Chung 2927* (HAST); *Pr. huangii* F.Wen & Z.B.Xin (≡ *Pr. repanda*), MK625449/MK625697/MK625699, China, Guangxi, Liuzhou, Liunan, Taiyangcun, *F. Wen WF150822-01* (IBK)*; *Pr. jianghuaensis* K.M.Liu & X.Z.Cai (≡ *Pr. bipinnatifida*), MK369982/MK369997/MK370008, China, Hunan, Jianghua, Daxu, Gaozhai, *K.-F. Chung 2932* (HAST)*; *Pr. jiangyongensis* X.L.Yu & MingLi, KY394902/KY393496/-- (Kong et al. 2017); *Pr. jingxiensis* (YanLiu, W.B.Xu & H.S.Gao) W.B.Xu & K.F.Chung, JX506907/JX506799/JX507004, China, Guangxi, Jingxi, Xinjing, Jinlong, *K.-F. Chung 1830* (HAST)*; *Pr. juliae* (Hance) Mich.Möller & A.Weber, KY394905/KY393499/-- (Kong et al. 2017); *Pr. langshanica* (W.T.Wang) Y.Z.Wang, KY394907/KY393501/-- (Kong et al. 2017); *Pr. latinervis* (W.T.Wang) Mich.Möller & A.Weber, KY394908/KY393502/-- (Kong et al. 2017); *Pr. laxiflora* (W.T.Wang) Y.Z.Wang, JX506910/JX506802/JX507007, China, Guangxi, Longzhou, Nonggang National Reserve, *C.-I Peng 22927* (HAST); *Pr. lechangensis* X.Hong, F.Wen & S.B.Zhou, KY394910/KY393504/-- (Kong et al. 2017); *Pr. leeii* (F.Wen, YueWang & Q.X.Zhang) Mich.Möller & A.Weber, MK369983/MK369998/--, China, Guangxi, Liuzhou, *K.-F. Chung 4154* (HAST); *Pr. leiophylla* (W.T.Wang) Y.Z.Wang, KY394912/KY393506/-- (Kong et al. 2017); *Pr. lepingensis* Z.L.Ning & M.Kang, KY394913/KY393507/-- (Kong et al. 2017); *Pr. leprosa* (YanLiu & W.B.Xu) W.B.Xu & K.F.Chung, JX506861/JX506750/JX507008, China, Guangxi, *W.-B. Xu et al. 091681* (IBK); *Pr. leyeensis* ined., MK369979/MK369994/MK370001, China, Guangxi, Leye, *K.-F. Chung 4193* (HAST); *Pr. liboensis* (W.T.Wang & D.Y.Chen) Mich.Möller & A.Weber, JX506911/JX506803/JX507010, *W.-B. Xu s.n.* (IBK); *Pr. liguliformis* (W.T.Wang) Mich.Möller & A.Weber, JX506912/JX506804/JX507011, *B. Pan s.n.* (IBK); *Pr. lijiangensis* (B.Pan & W.B.Xu) W.B.Xu & K.F.Chung, KY394919/KY393513/-- (Kong et al. 2017); *Pr. linearicalyx* F.Wen, B.D.Lai & Y.G.Wei, MH032854/MH032841/MH032846, China, Guangxi, Wuming, *W.-B. Xu et al. 12549* (IBK) (HAST); *Pr. linearifolia* (W.T.Wang) Y.Z.Wang, JX506913/JX506805/JX507012, China, Guangxi, Wuming, *W.-B. Xu et al. 12547* (IBK); *Pr. lingchuanensis* (YanLiu & Y.G.Wei) Mich.Möller & A.Weber (≡ *Pr. bipinnatifida*), JX506914/JX506806/JX507013, China, Guangxi, Lingchuan, Dajing, Pingshan, *K.-F. Chung 1802* (HAST)*, MK503715/--/--, *K.-F. Chung 3029* (HAST)*; *Pr. linglingensis* (W.T.Wang) Mich.Möller & A.Weber, MK369980/MK369995/MK370009, China, Guangxi, Quanzhou, *W.-B. Xu et al. 10021* (IBK); *Pr. linglingensis* var. *fragrans* F.Wen, Y.Z.Ge & B.Pan, MK369984/MK369999/MK370010, China, Guangxi, Quanzhou, *B.Pan 130409-01* (HAST); *Pr. liujiangensis* (D.Fang & D.H.Qin) YanLiu, KY394924/KY393518/-- (Kong et al. 2017); *Pr. lobulata* (W.T.Wang) Mich.Möller & A.Weber, JX506915/JX506807/JX507015, China, Guangdong, Yangshan, *W.-B. Xu et al. 11793* (IBK)*; *Pr. longgangensis* (W.T.Wang) YanLiu & Y.Z.Wang, JX506863/JX506752/JX507016, China, Guangxi, Longzhou, *Y.-S. Huang et al. Y3133* (HAST); *Pr. longicalyx* (J.M.Li & Y.Z.Wang) Mich.Möller & A.Weber, KY394927/KY393521/-- (Kong et al. 2017); *Pr. longii* (Z.Y.Li) Z.Y.Li, JX506917/JX506809/JX507019, China, Guangxi, Yongfu, *W.-B. Xu s.n.* (IBK); *Pr. longzhouensis* (B.Pan & W.H.Wu) W.B.Xu & K.F.Chung, JX506918/JX506810/JX507020, China, Guangxi, Longzhou, Shuikuo, *C.-I Peng 22963* (HAST)*; *Pr. lunglinensis* (W.T.Wang) Mich.Möller & A.Weber, KY394930/ KY393524/-- (Kong et al. 2017); *Pr. lungzhouensis* (W.T.Wang) Mich.Möller & A.Weber, KY394931/KY393525/-- (Kong et al. 2017); *Pr. luochengensis* (YanLiu & W.B.Xu) Mich.Möller & A.Weber, JX506920/JX506812/JX507022, China, Guangxi, Luocheng, *W.-B. Xu et al. 06044* (IBK); *Pr. lutea* (YanLiu & Y.G.Wei) Mich.Möller & A.Weber, JX506921/JX506813/JX507023, China, Guangxi, Cangwu, Shiqiao, *K.-F. Chung 1844* (HAST)*; *Pr. lutescens* B.Pan & H.S.Ma, MK369977/MK369992/MK370011, China, Guangxi, Lingshan, *B. Pan et al. H.-S. Ma MHS2016080401* (IBK); *Pr. lutvittata* F.Wen & Y.G.Wei, MK369978/MK369993/MK370012, China, Guangdong, Yangchun, *W.-B. Xu 11855* (IBK); *Pr. luzhaiensis* (YanLiu, Y.S.Huang & W.B.Xu) Mich.Möller & A.Weber, KC190197/KC190204/KC190211, China, Guangxi, Luzhai, *H.-Y. Huang 19* (HAST); *Pr. mabaensis* K.F.Chung & W.B.Xu, KY786298/KY786306/KY786318, China, Guangdong, Shaoguan, Qujiang, Maba, *C.-I Peng 24011* (HAST); *Pr. maciejewskii* F.Wen, R.L.Zhang & A.Q.Dong, KY394992/KY393623/-- (Kong et al. 2017); *Pr. macrodonta* (D.Fang & D.H.Qin) Mich.Möller & A.Weber, JX506924/JX506816/JX507026, China, Guangxi, Lingchuan, *W.-B. Xu s.n.* (IBK); *Pr. macrorhiza* (D.Fang & D.H.Qin) Mich.Möller & A.Weber, --/MK370000/MK370013, China, Guangxi, Guigang, *W.-B. Xu 11248* (IBK); *Pr. maculata* W.B.Xu & J.Guo, KU220604/KU220609/KU220605, China, Guangdong, Yangchun, Shiwang, *W.-B. Xu 11916* (IBK); *Pr. malipoensis* L.H.Yang & M.Kang, KY395003/ KY393633/-- (Kong et al. 2017); *Pr. medica* (D.Fang ex W.T.Wang) Y.Z.Wang, JX506864/JX506753/JX507027, China, Guangxi, Pingle, Qinglong, Lungshan, *K.-F. Chung 1850* (HAST); *Pr. minor* F.Wen & Y.G.Wei, MH032855/MH032840/MH032847, China, Hunan, Daoxian, Yueyan, *K.-F. Chung 2939* (HAST); *Pr. minutimaculata* (D.Fang & W.T.Wang) Y.Z.Wang, JX506865/JX506754/JX507029, China, Guangxi, Longzhou, *Y.-S. Huang H09733* (IBK); *Pr. moi* F.Wen & Y.G.Wei, KY394942/KY393536/-- (Kong et al. 2017); *Pr. mollifolia* (D.Fang & W.T.Wang) J.M.Li & Y.Z.Wang, JX506866/JX506755/JX507031, China, Guangxi, Yizhou, Beiya, Baowei, Machao, *K.-F. Chung 1819* (HAST)*; *Pr. multifida* B.Pan & K.F.Chung, JX506927/JX506756/JX507032, China, Guangxi, Yangshuo, Gaotian, Paitou, *K.-F. Chung 1853* (HAST)*; *Pr. nandanensis* (S.X.Huang, Y.G.Wei & W.H.Luo) Mich.Möller & A.Weber, JX506929/JX506820/JX507034, China, Guangxi, Nandan, *W.-B. Xu 09521* (IBK); *Pr. napoensis* (Z.Y.Li) Mich.Möller & A.Weber, JX506930/JX506821/JX507035, China, Guangxi, Napo, *W.-B. Xu et al. 10022* (IBK); *Pr. ningmingensis* (YanLiu & W.H.Wu) W.B.Xu & K.F.Chung, JX506931/JX506822/JX507036, China, Guangxi, Ningming, *W.-B. Xu et al.091737* (IBK); *Pr. obtusidentata* (W.T.Wang) Mich.Möller & A.Weber, KY394950/KY393544/-- (Kong et al. 2017); *Pr. ophiopogoides* (D.Fang & W.T.Wang) Y.Z.Wang, KY394951/KY393545/-- (Kong et al. 2017); *Pr. orthandra* (W.T.Wang) Mich.Möller & A.Weber, KF498147/KF498249/-- (Kang et al. 2014); *Pr. parvifolia* (W.T.Wang) Y.Z.Wang & J.M.Li, KY394952/KY393546/-- (Kong et al. 2017); *Pr. pengii* W.B.Xu & K.F.Chung, KY786302/KY786310/KY786322, China, Guangdong, Shaoguan, Ruyuan, Luoyang, *C.-I Peng 24024* (HAST); *Pr. petrocosmeoides* Bo Pan & F.Wen, MK369972/MK369987/MK370014, China, Guangxi, Jingxi, *B. Pan et al. 091696* (IBK); *Pr. pinnata* (W.T.Wang) Y.Z.Wang, JX506867/JX506757/JX507034, China, Guangxi, *K.-F. Chung 1873* (HAST); *Pr. pinnatifida* (Hand.-Mazz.) Y.Z.Wang, JX506868/JX506758/JX507035, China, Guangxi, *W.-B. Xu et al. 09408* (IBK); *Pr. polycephala* (Chun) Mich.Möller & A.Weber, KY786297/KY786307/KY786319, China, Guangdong, Shaoguan, Qujiang, Luokeng, Hewu, *C.-I Peng 24000* (HAST); *Pr. porphyria* X.L.Yu & MingLi, MH032856/MH032839/MH032848, China, Hunan, Yongzhou, Dong'an, *X.-L. Yu 140604* (CSFI); *Pr. pseudoeburnea* (D.Fang & W.T.Wang) Mich.Möller & A.Weber, MH032857/MH032838/MH032849, China, Guangxi, Tiandong, *B. Pan PR20120515* (IBK); *Pr. pseudoheterotricha* (T.J.Zhou, B.Pan & W.B.Xu) Mich.Möller & A.Weber, JX506933/JX506824/JX507041, China, Guangxi, Zhongshan, *W.-B. Xu et al. 09803* (IBK); *Pr. pseudolinearifolia* W.B.Xu & K.F.Chung, KY394933/KY393527/-- (Kong et al. 2017); *Pr. pseudomollifolia* W.B.Xu & YanLiu, JX506869/JX506759/JX507042, China, Guangxi, Rongshui, *K.-F. Chung 1810* (HAST)*; *Pr. pseudoroseoalba* JianLi, F.Wen & L.J.Yan, KY394959/KY393553/-- (Kong et al. 2017); *Pr. pteropoda* (W.T.Wang) YanLiu, KY394960/KY393554/-- (Kong et al. 2017); *Pr. pungentisepala* (W.T.Wang) Mich.Möller & A. Weber, JX506937/JX506828/JX507047, China, Guangxi, Longzhou, *W.-B. Xu et al. 12538* (IBK); *Pr. purpurea* F.Wen, BoZhao & Y.G.Wei, KY394964/KY393558/-- (Kong et al. 2017); *Pr. qingyuanensis* Z.L.Ning & M.Kang, KY394965/KY393559/-- (Kong et al. 2017); *Pr. renifolia* (D.Fang & D.H.Qin) J.M.Li & Y.Z.Wang, JX506737/JX506851/JX507048, China, Guangxi, Du'an, Xia'ao, Guanglong, Baiyan Dong, *W.-C. Leong 4072* (HAST); *Pr. repanda* (W.T.Wang) Y.Z.Wang, JX506940/JX506831/JX507054, China, Guangxi, Liujiang, Baipeng, Xiaoshang, *C.-I Peng 22921* (HAST), JX506938/JX506829/JX507051, Guangxi, Tian’e, Bamu, Dulou, Nalang, *K.-F. Chung 1821* (HAST)*, JX506873/JX506764/JX507052, Guangxi, Tian’e, Bamu, Dulou, Lahaoyan, *K.-F. Chung 1823* (HAST), JX506872/JX506763/JX507050, Rongshui, Rongshui, Guding, *K.-F. Chung 1815* (HAST); *Pr. repanda* var. *guilinensis* (W.T.Wang) Mich.Möller & A.Weber [≡ *Pr. subulata* var. *guilinensis* (W.T.Wang) W.B.Xu & K.F.Chung, comb. nov.], JX506941/JX506832/JX507055, China, Guangxi, Guiling, Qixing Park, Big Dipper Rock, *K.-F. Chung 1806* (HAST)*, JX506939/JX506830/JX507053, China, Guangxi, Hezhou, *K.-F. Chung 1845* (HAST); *Pr. ronganensis* (D.Fang & Y.G.Wei) Mich.Möller & A.Weber, JX506942/JX506833/JX507056, China, Guangxi, Rong'an, *W.-B. Xu s.n.* (IBK); *Pr. rongshuiensis* (YanLiu & Y.S.Huang) W.B.Xu & K.F.Chung, KY394971/KY393565/-- (Kong et al. 2017); *Pr. roseoalba* (W.T.Wang) Mich.Möller & A.Weber, KY394972/KY393566/-- (Kong et al. 2017); *Pr. rosulata* (F.Wen & Y.G.Wei) Z.L.Ning & X.Y.Zhuang, KR061643/--/-- (Kong et al. 2017); *Pr. rubella* L.H.Yang & M.Kang, KY394976/KY393570/-- (Kong et al. 2017); *Pr. rubribracteata* Z.L.Ning & M.Kang, MH032858/MH032837/MH032850, China, *Z.-L. Ning & M. Kang. N150426* (IBSC); *Pr. sclerophylla* (W.T.Wang) YanLiu, JX506943/JX506834/JX507057, China, Guangxi, Du'an, *W.-B. Xu et al. 09337* (IBK); *Pr. shouchengensis* (Z.Y.Li) Z.Y.Li, JX506944/JX506835/KC190215, China, Guangxi, Yongfu, *W.-B Xu s.n.* (IBK); *Pr. sinovietnamica* W.H.Wu & Q.Zhang, MK369973/MK369988/MK370015, Vietnam, Vietnam. Lang Son, Huu Lung District, Huu Lien Commune, Tam Lai Village, *C.-I Peng 21956* (HAST); *Pr. spadiciformis* (W.T.Wang) Mich.Möller & A.Weber, FJ501346/AJ492291/-- (Kong et al. 2017); *Pr. spinulosa* (D.Fang & W.T.Wang) Y.Z.Wang, JX506948/JX506839/JX507062, China, Guangxi, Fusui, *W.-B. Xu et al. 12545* (IBK); *Pr. subrhomboidea* (W.T.Wang) Y.Z.Wang, JX506949/JX506840/JX507064, China, Guangxi, Lingchuan, *W.-B. Xu et al. 12030* (IBK); *Pr. subulata* (W.T.Wang) Mich.Möller & A.Weber, KY395020/KY393579/-- (Kong et al. 2017); *Pr. subulata* var. *yangchunensis* (W.T.Wang) Mich.Möller & A.Weber, KY786290/KY786314/KY786326, China, Guangdong, Yangchun, Tanshui, Fenglai, *C.-I Peng 23959* (HAST)*; *Pr. suichuanensis* X.L.Yu & J.J.Zhou, KY395021/KY393580/-- (Kong et al. 2017); *Pr. swinglei* (Merr.) Mich.Möller & A.Weber, JX506950/JX506841/JX507065, China, Guangxi, Rongxian, *W.-B. Xu et al. 12124* (IBK); *Pr. tabacum* Hance, JX506875/JX506766/JX507066, China, Guangxi, Hezhou, Lingfengshan, Xiannvdong, *K.-F. Chung 1846* (HAST); *Pr. tenuifolia* (W.T.Wang) Y.Z.Wang, KY395024/KY393583/-- (Kong et al. 2017); *Pr. tenuituba* (W.T.Wang) Y.Z.Wang, KY395025/KY393584/-- (Kong et al. 2017); *Pr. tiandengensis* (F.Wen & H.Tang) F.Wen & K.F.Chung, KY395027/KY393586/-- (Kong et al. 2017); *Pr. tribracteata* (W.T.Wang) Mich.Möller & A.Weber, JX506951/JX506842/JX507067, China, Guangxi, Fengshan, *H.-S. Gao 07123* (IBK); *Pr. tribracteata* var. *zhuana* (Z.Y.Li, Q.Xing & Y.B.Li) Mich.Möller & A.Weber, JX506952/JX506843/JX507068, China, Guangxi, *K.-F. Chung 1877* (HAST); *Pr. tsoongii* H.L.Liang, BoZhao & F.Wen, KY395029/KY393588/-- (Kong et al. 2017); *Pr. varicolor* (D.Fang & D.H.Qin) Y.Z.Wang, KY395030/ KY393589/-- (Kong et al. 2017); *Pr. verecunda* (Chun) Mich.Möller & A.Weber, KY395031/KY393590/-- (Kong et al. 2017); *Pr. villosissima* (W.T.Wang) Mich.Möller & A.Weber, KY395032/KY393591/-- (Kong et al. 2017); *Pr. weii* Mich.Möller & A.Weber, DQ872832/DQ872811/-- (Li and Wang 2007); *Pr. wentsaii* (D.Fang & L.Zeng) Y.Z.Wang, JX506953/JX506844/JX507069, China, Guangxi, Longzhou, *Y.-S. Huang et al. Y1314* (IBK); *Pr. xinningensis* (W.T.Wang) Mich.Möller & A.Weber, KY395035/KY393594/-- (Kong et al. 2017); *Pr. xiuningensis* (X.L.Liu & X.H.Guo) Mich.Möller & A.Weber, JX506954/JX506845/JX507074, Dr. Cecilia Koo Botanic Conservation Center; *Pr. xiziae* F.Wen, YueWang & G.J.Hua, KY395038/KY393597/-- (Kong et al. 2017); *Pr. yangchunensis* Y.L.Zheng & Y.F.Deng, MH032859/MH032836/MH032851, China, Guangdong,Yangchun, *W.-B. Xu et al. 11856* (IBK); *Pr. yangshanensis* W.B.Xu & B.Pan, JX506874/JX506765/JX507060, China, Guangdong, Qingyuan, Yangshan, Qinglian, Tangliao, *K.-F. Chung 1835* (HAST); *Pr. yangshuoensis* Y.G.Wei & F.Wen, JX506955/JX506846/JX507070, China, Guangxi, Yangshuo, *W.-B. Xu et al. 091750* (IBK); *Pr. yingdeensis* Z.L.Ning, M.Kang & X.Y.Zhuang, KY395043/KY393602/-- (Kong et al. 2017); *Pr. yungfuensis* (W.T.Wang) Mich.Möller & A.Weber, JX506958/JX506849/JX507073, China, Guangxi, *W.-B. Xu s.n.* (IBK); *Pr. zhoui* F.Wen & Z.B.Xin [≡ *Pr. bipinnatifida* var. *zhoui* (F.Wen & Z.B.Xi) W.B.Xu & K.F.Chung, comb. & stat. nov.], MK625450/MK625698/MK625700, China, Guangxi, Liujiang, Liyong, *F. Wen* *WF150718-01* (IBK)*.

**References**

Kang M, Tao J, Wang J, Ren C, Qi Q, Xiang Q-Y, Huang H. 2014. Adaptive and nonadaptive genome size evolution in Karst endemic flora of China. New Phytol 202(4):1371-1381. https://doi.org/10.1111/nph.12726

Kong HH, Condamine FL, Harris AJ, Chen JL, Pan B, Möller M, Hoang VS, Kang M. 2017. Both temperature fluctuations and East Asian monsoons have driven plant diversification in the karst ecosystems from southern China. Mol Ecol 26(22):6414-6429. https://doi.org/10.1111/mec.14367

Li J-M, Wang Y-Z. 2007. Phylogenetic reconstruction among species of *Chiritopsis* and *Chirita* sect. *Gibbosaccus* (Gesneriaceae) based on nrDNA ITS and cpDNA *trnL-F* sequences. Syst Bot 32(4):888-898. https://doi.org/10.1600/036364407783390764

Möller M, Pfosser M, Jang CG, Mayer V, Clark A, Hollingsworth ML, Barfuss MHJ, Wang YZ, Kiehn M, Weber A. 2009. A preliminary phylogeny of the 'Didymocarpoid Gesneriaceae' based on three molecular data sets: incongruence with available tribal classifications. Am J Bot 96(5):989-1010. https://doi.org/10.3732/Ajb.0800291

Xu W-B, Liu Y, Kono Y, Chang H, Peng C-I, Chung K-F. 2013. *Pr. cardaminifolia* (Gesneriaceae), a rare new species from limestone areas in Guangxi, China. Bot Stud 24:19. https://doi.org/doi:10.1186/1999-3110-54-19
